# The stringent response regulator (p) ppGpp mediates virulence gene expression and survival in *Erwinia amylovora*

**DOI:** 10.1186/s12864-020-6699-5

**Published:** 2020-03-30

**Authors:** Ho-wen Yang, Menghao Yu, Jae Hoon Lee, Tiyakhon Chatnaparat, Youfu Zhao

**Affiliations:** Department of Crop Sciences, University of Illinois at Urbana-Champaign, 1201 W. Gregory Dr, Urbana, IL 61801 USA

**Keywords:** *Erwinia amylovora*, RNA-seq, (p) ppGpp, Virulence factors, T3SS

## Abstract

**Background:**

The nucleotide second messengers, i.e., guanosine tetraphosphate and pentaphosphate [collectively referred to as (p) ppGpp], trigger the stringent response under nutrient starvation conditions and play an essential role in virulence in the fire blight pathogen *Erwinia amylovora*. Here, we present transcriptomic analyses to uncover the overall effect of (p) ppGpp-mediated stringent response in *E. amylovora* in the *hrp*-inducing minimal medium (HMM).

**Results:**

In this study, we investigated the transcriptomic changes of the (p) ppGpp^0^ mutant under the type III secretion system (T3SS)-inducing condition using RNA-seq. A total of 1314 differentially expressed genes (DEGs) was uncovered, representing more than one third (36.8%) of all genes in the *E. amylovora* genome. Compared to the wild-type, the (p) ppGpp^0^ mutant showed down-regulation of genes involved in peptide ATP-binding cassette (ABC) transporters and virulence-related processes, including type III secretion system (T3SS), biofilm, and motility. Interestingly, in contrast to previous reports, the (p) ppGpp^0^ mutant showed up-regulation of amino acid biosynthesis genes, suggesting that it might be due to that these amino acid biosynthesis genes are indirectly regulated by (p) ppGpp in *E. amylovora* or represent specific culturing condition used. Furthermore, the (p) ppGpp^0^ mutant exhibited up-regulation of genes involved in translation, SOS response, DNA replication, chromosome segregation, as well as biosynthesis of nucleotide, fatty acid and lipid.

**Conclusion:**

These findings suggested that in HMM environment, *E. amylovora* might use (p) ppGpp as a signal to activate virulence gene expression, and simultaneously mediate the balance between virulence and survival by negatively regulating DNA replication, translation, cell division, as well as biosynthesis of nucleotide, amino acid, fatty acid, and lipid. Therefore, (p) ppGpp could be a promising target for developing novel control measures to fight against this devastating disease of apples and pears.

## Background

During the early stage of infection, plant pathogenic bacteria are exposed to environmental stresses, including nutrient starvation and oxidative stress. To overcome these adverse conditions, bacteria produce linear nucleotide second messengers, i. e. guanosine tetraphosphate and pentaphosphate [collectively referred to as (p) ppGpp], to regulate gene expression from replication and growth to colonization and survival [1]. This phenomenon is so-called the stringent response, one of the global regulatory systems in bacteria [1]. Biosynthesis of (p) ppGpp is mainly attributed to the RelA/SpoT homologue proteins (RSH). RelA is a ribosomal associated protein which synthesizes (p) ppGpp in response to amino acid starvation. On the other hand, SpoT is a dual function protein which synthesizes (p) ppGpp in response to fatty acid, carbon, phosphorous, and iron limitations, and also degrades (p) ppGpp to prevent replication halt due to high concentration of (p) ppGpp [1–4]. It has been reported that the *relA/spoT* double mutant resulted in multiple defects, including autotrophies for several amino acids [5].

Several models have been proposed for the molecular mechanisms of the stringent response [1, 2, 6]. It has been reported that (p) ppGpp, along with a small RNA polymerase (RNAP) binding protein DksA, directly binds to RNAP and then destabilizes its open complex [6, 7]. On the other hand, (p) ppGpp regulates gene expression indirectly by sigma factor competition [2]. High concentration of (p) ppGpp inhibits sigma factor σ^70^, which allows more free RNAPs interact with alternative sigma factors, including σ^54^, to activate genes in response to stresses [3, 8]. Moreover, (p) ppGpp also influences gene expression other than through RNAP [9] by directly down-regulating stable RNA (rRNA and tRNA) and genes related to transcription and translation, while directly up-regulating amino acid biosynthesis genes [1, 3, 8, 10, 11]. It has been reported that over 30% genes in *Escherichia coli* genome were differentially expressed by (p) ppGpp, including up-regulation of genes related to stress response and down-regulation of genes related to macromolecular structures in isoleucine starvation condition [12]. About 500 genes were found to be differentially expressed in *E. coli* strain MG1655 under serine hydroxamate (SHX) treatment, which mimics serine starvation [13].

Previous studies showed that (p) ppGpp is required for virulence gene expression in *Salmonella enterica* [14], *E. coli* [15], *Pseudomonas syringae* [16], and *Erwinia amylovora* [17]. *E. amylovora* is the causal agent of the fire blight disease, a devastating disease that causes severe economic losses in apples and pears [18]. One of the major pathogenicity factors in *E. amylovora* is the hypersensitive response and pathogenicity (*hrp*)-type III secretion system (T3SS) [19]. The alternative sigma factor HrpL is the master regulator of T3SS, which in turn is activated by another alternative sigma factor 54 (RpoN), along with several other proteins, including HrpS, IHF, and YhbH [20–23]. Previous study has demonstrated that (p) ppGpp activates the RpoN and HrpL sigma factor cascade to trigger the T3SS gene expression. Furthermore, a recent study showed that (p) ppGpp activates expression of a two-component system HrpXY, which in turn regulates the expression of the *hrpS* gene [23]. In this study, we investigated transcriptomic profiles of the wild-type strain (WT) and the (p) ppGpp^0^ mutant at 3 h post incubation (hpi), and we also compared global gene expression between WT grown at 3 and 6 hpi in HMM.

## Results and discussion

### Overview of the global effect of (p) ppGpp in gene expression in *Erwinia amylovora*

The linear nucleotide second messengers (p) ppGpp have been studied for more than four decades [13]. Based on previous reports, (p) ppGpp swiftly and robustly mediates target gene expression, such as genes related to transcription [24] and translation [25, 26]. Consequently, bacteria growth [3], surface organelle production (fimbriae and flagella) [27], cell size, and virulence [28] are affected. In this study, the global effect of (p) ppGpp in *E. amylovora* on gene expression was examined using RNA-seq. In summary, 13,167,843 to 15,637,863 reads for each biological sample were generated for *E. amylovora* WT and its (p) ppGpp^0^ mutant at 3 h, and the percentage of reads mapped to *E. amylovora* genome ranged from 97.1 to 97.8%; whereas 15,618,174 to 17,669,201 reads for each biological sample were obtained for *E. amylovora* WT at 6 h, and the percentage of reads mapped to *E. amylovora* genome were from 97.2 to 97.6%.

**Fig. 1:**
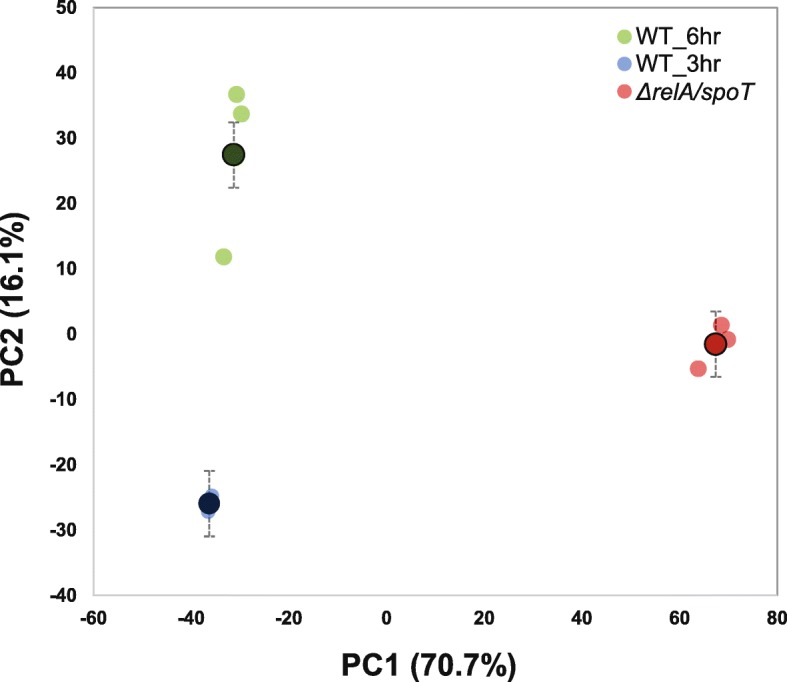
Principal component analysis (PCA) for characterization of gene expression dynamics in WT at 3 h and 6 h, as well as the (p) ppGpp^0^ mutant (*relA/spoT*) at 3 h in the *hrp*-inducing minimal medium

The gene expression dynamics was first characterized by principal component analysis (PCA) for substantially expressed genes (log_2_CPM ≥ 2 in at least 3 samples, CPM: counts per million reads) (Fig. 1). PC1 and PC2 explained 70.7 and 16.1% of the total variability, respectively. PC1 mainly explained the variability between WT and the (p) ppGpp^0^ mutant (*P* < 0.01), indicating that gene expression patterns were changed dramatically in the (p) ppGpp^0^ mutant. On the other hand, PC2 mainly explained the variability of gene expression at different time point for WT at 3 h and 6 h (*P <* 0.01). The PCA plot also showed obvious separation of the WT at 3 and 6 h as well as the (p) ppGpp^0^ mutant strain at 3 h. Nevertheless, three biological samples for each treatment were mostly clustered together, indicating excellent sample repetition (Fig. 1**).**

**Fig. 2:**
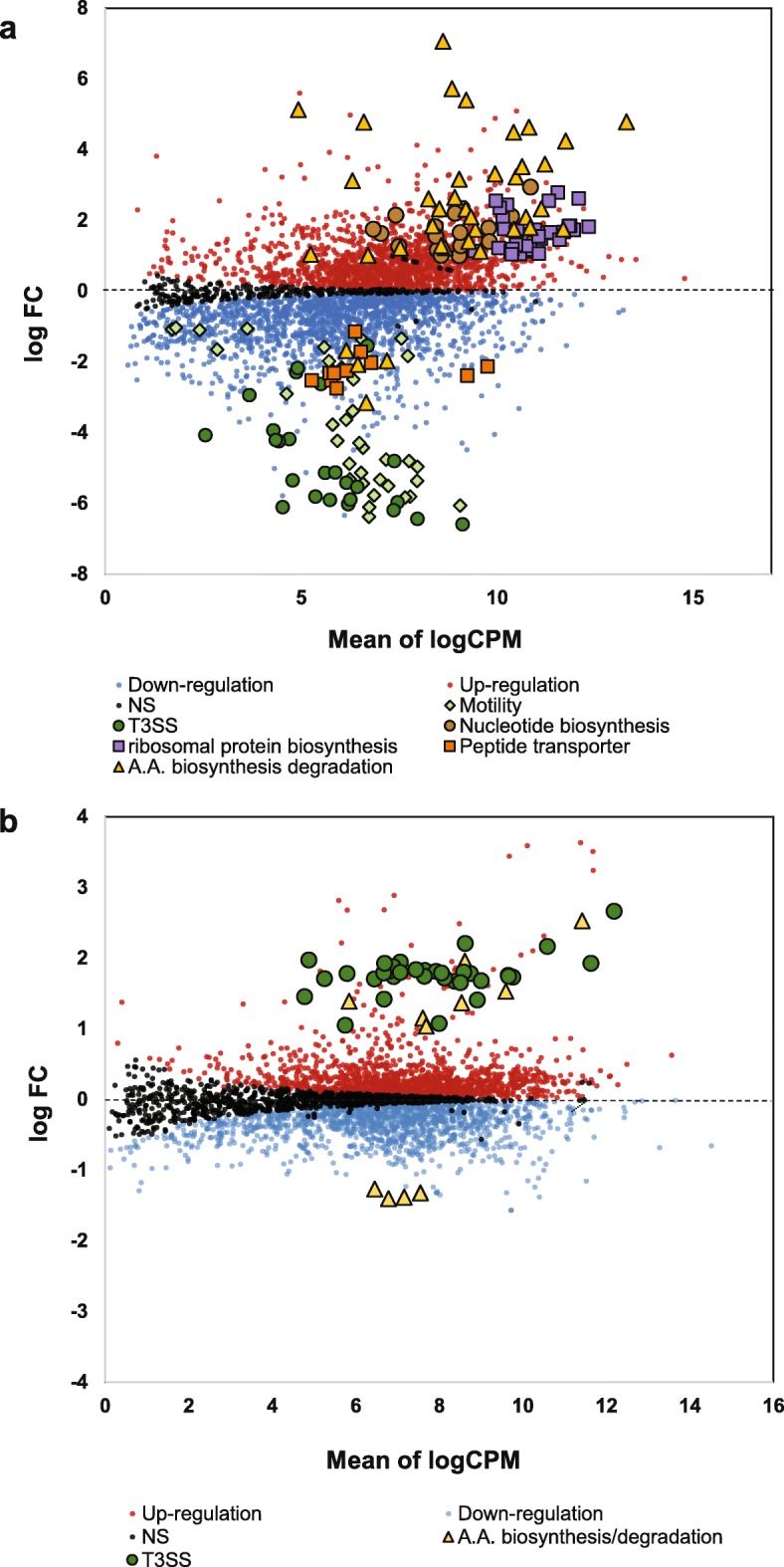
Identification of differentially expressed genes (DEGs) between the (p) ppGpp^0^ mutant and WT by quasi-likelihood (QL) F-test in edgeR. **a** Expression level and fold change of each gene by comparing the (p) ppGpp^0^ mutant versus WT at 3 h. The X and Y axes correspond to mean of normalized log2-based count per million values (log_2_CPM) and log_2_((p) ppGpp^0^/WT at 3 h) ratio, respectively. **b** Expression level and fold change of each gene by comparing the WT at 6 h versus WT at 3 h. The X and Y axes correspond to mean of normalized log2-based count per million values (log_2_CPM) and log_2_(WT at 6 h /WT at 3 h) ratio, respectively

**Fig. 3:**
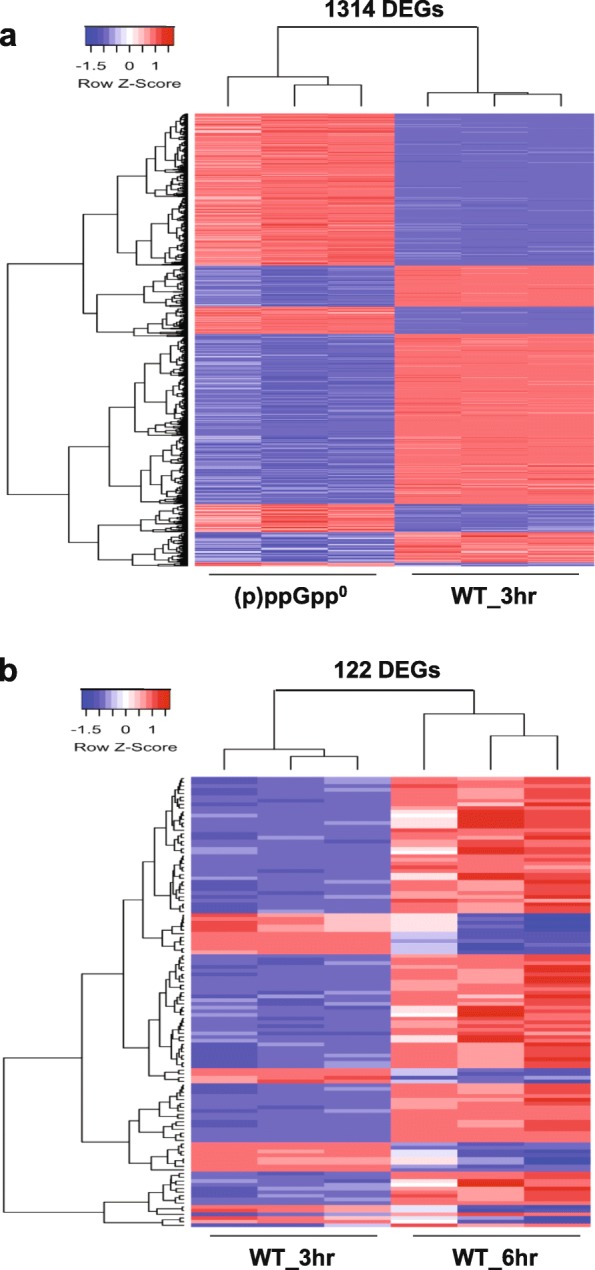
Heatmapt showing expression pattern of differentially expressed genes in three biological samples each for (**a**) the (p) ppGpp^0^ mutant and WT at 3 h (**b**) WT at 6 h and WT at 3 h. White represents mean of expression level (log_2_CPM), dark blue represents minimal gene expression, and bright red represents maximal gene expression

**Fig. 4:**
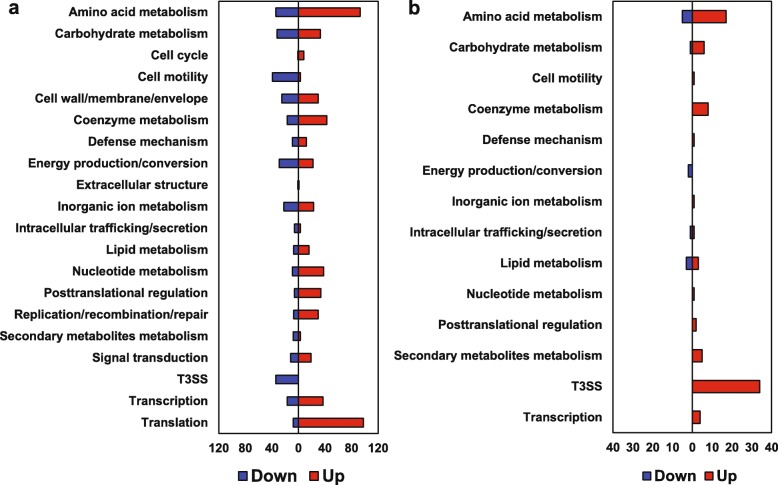
Classification and verification of differentially expressed genes (DEGs). Functional categories of DEGs according to the clusters of orthologous groups (COG) database. **a** the (p) ppGpp^0^ mutant and WT at 3 h (**b**) WT at 6 h and WT at 3 h. Red: up-regulated; blue: down-regulated

For analyzing genes that might be (p) ppGpp-dependent, DEGs were identified by comparing the (p) ppGpp^0^ mutant with WT at 3 h. A total of 1314 DEGs were identified, representing more than one third of genes (36.8%) in the *E. amylovora* genome. Among them, 612 DEGs (46.6%) were up-regulated and 702 DEGs (53.4%) were down-regulated in the (p) ppGpp^0^ mutant (Fig. 2a and Fig. 3a, Additional file 1: Table S1). Most DEGs were functionally categorized according to the clusters of orthologous groups (COG) (Fig. 4a). Most of the DEGs categorized as amino acid metabolism, coenzyme metabolism, translation, posttranslational regulation, replication/ recombination/DNA repair, as well as nucleotide metabolism, were negatively regulated by (p) ppGpp. Conversely, most of the DEGs categorized as T3SS, cell motility, and energy production/conversion were positively regulated by (p) ppGpp (Fig. 4a). These results supported the dogma that (p) ppGpp globally regulates gene expression.

**Fig. 5:**
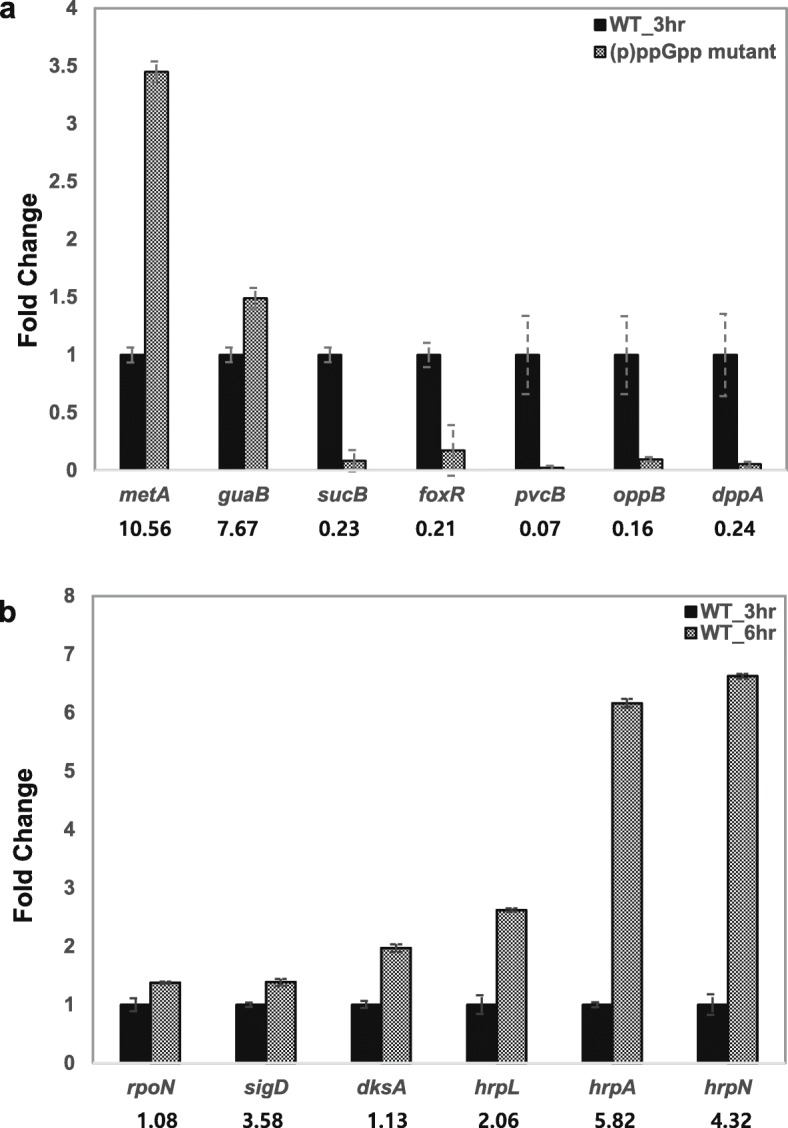
Validation of RNA-seq data by qRT-PCR. **a** the (p) ppGpp^0^ mutant and WT at 3 h (**b**) WT at 6 h and WT at 3 h. Numbers on the bar indicated fold changes obtained for the gene in RNA-seq

On the other hand, to investigate the hierarchical natural of response over time in HMM, we identified the DEGs between WT at 6 h and WT at 3 h. A total of 122 DEGs were identified, where 97 DEGs (87.4%) were up-regulated, and 14 (12.6%) DEGs were down-regulated in WT at 6 h (Fig. 2b and Fig. 3b). The majority of up-regulated genes are T3SS (*n* = 34) and amino acid metabolism (*n* = 17) (Fig. 4b, Additional file 2: Table S2), suggesting that after activation by (p) ppGpp, the expression of the T3SS genes was higher at 6 h as reported previously [23]. To verify the result of RNA-seq, qRT-PCR was conducted for several randomly selected DEGs, and the results of qRT-PCR were mostly in the similar trend as the RNA-seq data (Fig. 5a and b). In addition, expression of T3SS genes was previously verified [17].

### Positive regulation of virulence-related genes by (p) ppGpp

**Fig. 6:**
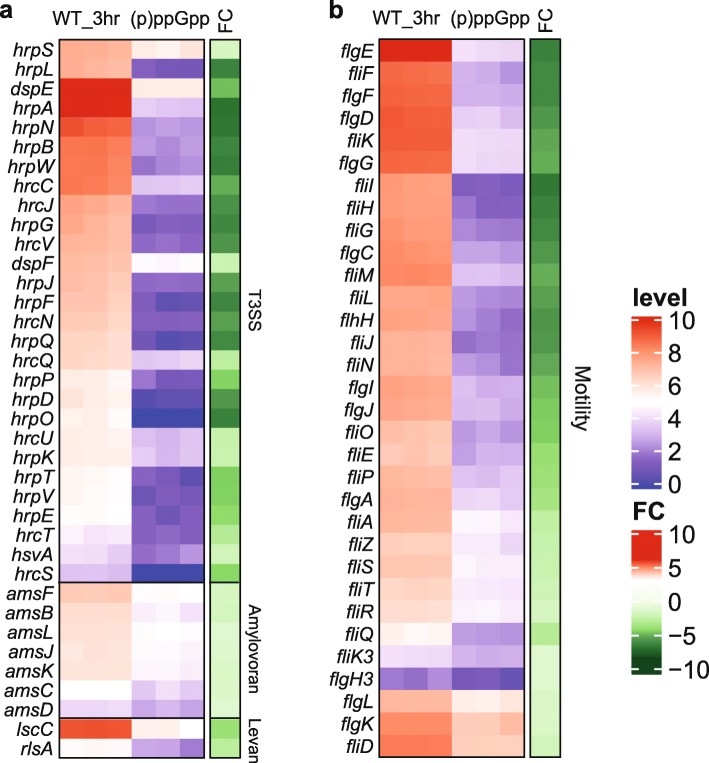
Differentially expressed genes positively regulated by (p) ppGpp. **a** T3SS, amylovoran, and levan. **b** motility-related genes. White represents mean of expression level (log_2_CPM), dark blue represents minimal gene expression, and bright red represents maximal gene expression. In the side bar (right), dark green represents higher negative fold change (log_2_FC), and bright red represents the higher positive log_2_FC

During the early stage of infection when bacteria are subjected to stress response, such as nutrient limitation and oxidative stress, (p) ppGpp is produced [17]. Previous research revealed that (p) ppGpp activates T3SS to trigger virulence [17]. Consistent with this result, our RNA-seq data showed that (p) ppGpp positively regulates virulence gene expression in *E. amylovora*, including most of the T3SS, amylovoran biosynthesis and levan production genes (Fig. 6a and Table 1). Among the T3SS genes, the *hrpL*, *hrpA, hrpN* and *hrpW* gene expression exhibited a very high negative fold change (log_2_FC = − 6.02 to − 6.49). The deficiency of T3SS gene expression in the (p) ppGpp^0^ mutant indicated that (p) ppGpp is required for T3SS expression in *E. amylovora* [17]. Down-regulation of the T3SS genes, accompanied by attenuated virulence and reduced growth, was also reported in the *P. syringae* (p) ppGpp^0^ mutants [16]. Similar results were also reported in *Bordetella pertussis* in response to glutamine limitation [29] and in *E. coli* in response to nutrient starvation [15]. Previous studies have demonstrated that the T3SS gene expression in *E. amylovora* reached the highest level at 6 hpi in HMM [23]. We found that 34 out of 97 up-regulated DEGs in comparison of WT at 6 h and WT at 3 h belongs to T3SS. Both *hrpA* and *hrpN* exhibited up-regulation more than two folds in WT at 6 h (log_2_FC = 2.54 & 2.11, respectively) (Table 2), indicating that T3SS might be continuously expressed after activation by (p) ppGpp at 3 h.

**Table 1:** List of differentially expressed genes^a^ associated with virulence for the (p) ppGpp^0^ mutant versus WT at 3 h

Accession	Gene Description	Log_**2**_FC^**b**^	Mean^**c**^
**T3SS**			
*EAMY_0519*	*hrpK*, pathogenicity locus protein	−2.08	4.91
*EAMY_0520*	*hsvA*, Hrp-associated systemic virulence protein	−1.75	3.35
*EAMY_0525*	*hrcU*, type III secretion protein	−2.21	4.88
*EAMY_0526*	*hrcT*, type III secretion apparatus protein	−2.84	3.67
*EAMY_0527*	*hrcS*, type III secretion protein	−3.87	2.55
*EAMY_0529*	*hrcQ*, type III secretion system apparatus protein	−2.53	5.50
*EAMY_0530*	*hrpP*, type III secretion protein	−4.06	4.70
*EAMY_0531*	*hrpO*, type III secretion protein	−5.86	4.53
*EAMY_0532*	*hrcN*, type III secretion system ATPase	−5.01	5.61
*EAMY_0533*	*hrpQ*, type III secretion system protein	−5.65	5.37
*EAMY_0534*	*hrcV*, type III secretion inner-membrane protein	−5.3	6.15
*EAMY_0535*	*hrpJ*, type III secretion system protein	−5.00	5.88
*EAMY_0536*	*hrpL*, RNA polymerase sigma factor	−5.89	6.20
*EAMY_0539*	*hrpS*, sigma-54-dependent enhancer-binding protein	−1.45	6.69
*EAMY_0542*	*hrpA*, Hrp pili protein	−6.49	9.12
*EAMY_0543*	*hrpB*, type III secretion system protein	−5.86	7.46
*EAMY_0544*	*hrcJ*, type III secretion inner-membrane protein	−5.42	6.44
*EAMY_0545*	*hrpD*, type III secretion protein	−5.19	4.78
*EAMY_0546*	*hrpE*, type III secretion apparatus protein	−3.82	4.29
*EAMY_0547*	*hrpF*, type III secretion protein	−5.75	5.74
*EAMY_0548*	*hrpG*, type III secretion protein	−5.75	6.26
*EAMY_0549*	*hrcC*, type III secretion system outer membrane pore	−4.70	7.37
*EAMY_0550*	*hrpT*, type III secretion lipoprotein	−4.11	4.43
*EAMY_0551*	*hrpV*, type III secretion protein	−4.07	4.35
*EAMY_0552*	*hrpN*, harpin protein	−6.32	7.96
*EAMY_0553*	*orfA*, Tir chaperone family protein	−4.38	6.87
*EAMY_0554*	*orfB*, avirulence protein	−3.33	5.12
*EAMY_0555*	*orfC*, HrpW-specific chaperone	−4.98	6.33
*EAMY_0556*	*hrpW*, harpin protein	−6.07	7.36
*EAMY_0557*	*dspE*, Hrp secreted pathogenicity-like protein	−4.4	9.23
*EAMY_0558*	*dspF*, Hrp secreted pathogenicity-like protein	−2.05	6.23
*EAMY_0653*	*eop2*, type III effector	−3.34	6.98
*EAMY_3175*	*avrRpt2*, cysteine protease avirulence protein	−1.07	6.20
**Amylovora**			
*EAMY_2242*	*amsL*, amylovoran biosynthesis protein	−1.04	5.64
*EAMY_2243*	*amsK*, glycosyltransferase	−1.26	5.46
*EAMY_2244*	*amsJ*, exopolysaccharide biosynthesis protein	−1.10	5.47
*EAMY_2245*	*amsF*, exopolysaccharide biosynthesis protein	−1.48	6.10
*EAMY_2247*	*amsD*, Amylovoran biosynthesis glycosyltransferase	−1.03	3.53
*EAMY_2248*	*amsC*, exopolysaccharide biosynthesis protein	−1.18	4.53
*EAMY_2249*	*amsB*, glycosyltransferase	−1.62	5.59
**Levan**			
*EAMY_3695*	*lscC*, levansucrase	−3.60	8.19
*EAMY_0559*	*rlsA*, transcriptional regulator	−2.62	4.51

^a^Differentially expressed genes (DEGs) between the (p) ppGpp^0^ mutant and WT at 3 h with |log_2_FC| value ≥ 1 and an adjusted *p* value < 0.05. WT: wild type. FC: fold change. FC values below 0 mean that the gene has lower expression in the (p) ppGpp^0^ mutant than in WT.

^b^log_2_FC valus was calculated by the log based 2 value of (p) ppGpp^0^ / WT at 3 h

^c^The average of log_2_CPM was calculated. CPM: count per million reads

**Table 2:** List of differentially expressed genes^a^ associated with virulence for WT at 6 h versus WT at 3 h

Accession	Gene description	Log_**2**_FC^**b**^	Mean^**c**^
**T3SS**			
*EAMY_0519*	*hrpK*, pathogenicity locus protein	1.38	6.50
*EAMY_0520*	*hsvA*, Hrp-associated systemic virulence protein	1.6	4.98
*EAMY_0521*	*hsvB*, Hrp-associated systemic virulence protein	1.4	4.58
*EAMY_0522*	*hsvC*, Hrp-associated systemic virulence protein	1.02	5.64
*EAMY_0525*	*hrcU*, type III secretion protein	1.68	6.64
*EAMY_0526*	*hrcT*, type III secretion apparatus protein	1.71	5.51
*EAMY_0527*	*hrcS*, type III secretion protein	1.9	4.54
*EAMY_0528*	*hrcR*, type III secretion apparatus protein	1.75	6.51
*EAMY_0529*	*hrcQ*, type III secretion system apparatus protein	1.75	7.37
*EAMY_0530*	*hrpP*, type III secretion protein	1.83	6.74
*EAMY_0531*	*hrpO*, type III secretion protein	1.78	6.58
*EAMY_0532*	*hrcN*, type III secretion system ATPase	1.72	7.64
*EAMY_0533*	*hrpQ*, type III secretion system protein	1.67	7.37
*EAMY_0534*	*hrcV*, type III secretion inner-membrane protein	1.6	8.13
*EAMY_0535*	*hrpJ*, type III secretion system protein	1.63	7.86
*EAMY_0536*	*hrpL*, RNA polymerase sigma factor	1.04	7.89
*EAMY_0542*	*hrpA*, Hrp pili protein	2.54	11.62
*EAMY_0543*	*hrpB*, type III secretion system protein	1.67	9.49
*EAMY_0544*	*hrcJ*, type III secretion inner-membrane protein	1.7	8.47
*EAMY_0545*	*hrpD*, type III secretion protein	1.69	6.78
*EAMY_0546*	*hrpE*, type III secretion apparatus protein	1.65	6.19
*EAMY_0547*	*hrpF*, type III secretion protein	1.71	7.77
*EAMY_0548*	*hrpG*, type III secretion protein	1.71	8.30
*EAMY_0549*	*hrcC*, type III secretion system outer membrane porein	1.68	9.38
*EAMY_0550*	*hrpT*, type III secretion lipoprotein	1.71	6.39
*EAMY_0551*	*hrpV*, type III secretion protein	1.82	6.37
*EAMY_0552*	*hrpN*, harpin protein	2.11	10.21
*EAMY_0553*	*orfA*, Tir chaperone family protein	1.61	8.27
*EAMY_0554*	*orfB*, avirulence protein	1.64	8.76
*EAMY_0555*	*orfC*, HrpW-specific chaperone	1.75	7.15
*EAMY_0556*	*hrpW*, harpin protein	1.68	9.40
*EAMY_0557*	*dspE*, Hrp secreted pathogenicity-like protein	1.86	11.32
*EAMY_0558*	*dspF*, Hrp secreted pathogenicity-like protein	2.11	8.21
*EAMY_0653*	*eop2*, type III effector	1.36	8.74

^a^Differentially expressed genes (DEGs) between the WT at 6 h and at 3 h with |log_2_FC| value ≥ 1 and an adjusted *p* value < 0.05. FC: fold change. WT: wild type. FC values over 0 mean that the gene has higher expression in WT at 6 h than at 3 h

^b^log_2_FC valus was calculated by the log based 2 value of (p) ppGpp^0^ / WT at 3 h

^c^The average of log_2_CPM was calculated. CPM: count per million reads

In addition, levan and amylovoran are also virulence factors and contribute to biofilm formation in *E. amylovora* [30]. Diminished biofilm formation and attenuated virulence has been reported in the (p) ppGpp-deficient mutant of *E. coli* [31] and *Enterococcus faecalis* [32]. We found that both amylovoran biosynthesis (*amsBCDFJKL*) and levan production (*lscC* and *rlsA*) genes exhibited negative fold change (Fig. 6a and Table 1), indicating that (p) ppGpp positively regulates virulence by affecting most of the levan and amylovoran production genes.

Flagella-mediated motility is another important virulence determinant [33]. There are three classes of flagellar genes in hierarchical order: class I (*flhDC*, master regulator of flagellar formation), class II (hook and basal body formation), and class III (filament and motor assembly [34]. A defect in motility due to the loss of flagella in the (p) ppGpp^0^ mutant has been reported in *S. enterica* [35], *E. coli* [27], *P. syringae* [16], and *E. amylovora* (Additional file 3**:** Fig. S1). We found that 32 DEGs related to motility (Fig. 6b, Table 3) were differentially expressed between the (p) ppGpp^0^ mutant and the WT. Almost all DEGs belongs to class II, including *flgEFDC* and *fliFIHGLJ*, which exhibited the highest negative fold changes (− 6.1 ≤ |log_2_FC| ≤ − 5.3, − 5.1 ≤ |log_2_FC| ≤ − 6.1, respectively).

**Table 3:** List of differentially expressed genes^a^ associated with motility for the (p) ppGpp^0^ mutant versus WT at 3 h

Accession	Gene Description	Log_**2**_FC^**b**^	Mean^**c**^
*EAMY_1508*	*fliI*, flagellum-specific ATP synthase	−6.25	6.73
*EAMY_1509*	*fliH*, flagellar assembly protein	−5.98	6.74
*EAMY_1456*	*flgE*, flagellar hook protein	−5.97	9.06
*EAMY_1511*	*fliF*, flagellar M-ring protein	−5.73	7.65
*EAMY_1457*	*flgF*, flagellar basal body rod protein	−5.71	7.79
*EAMY_1510*	*fliG*, flagellar motor switch protein	−5.66	6.86
*EAMY_1453*	*flgB*, flagellar basal body protein	−5.4	7.22
*EAMY_1459*	*flhH*, flagellar L-ring protein	−5.33	6.57
*EAMY_1455*	*flgD*, flagellar hook capping protein	−5.26	7.96
*EAMY_1454*	*flgC*, flagellar basal body rod protein	−5.23	7.01
*EAMY_1507*	*fliJ*, flagellar biosynthesis chaperone	−5.22	6.24
*EAMY_1505*	*fliL*, flagellar basal body-associated protein	−5.03	6.53
*EAMY_1506*	*fliK*, flagellar hook-length control protein	−4.88	7.96
*EAMY_1503*	*fliN*, flagellar motor switch protein	−4.77	6.23
*EAMY_1458*	*flgG*, flagellar basal body rod protein	−4.71	7.75
*EAMY_1504*	*fliM*, flagellar motor switch protein	−4.66	7.17
*EAMY_1460*	*flgI*, flagellar P-ring protein	−4.33	6.58
*EAMY_1461*	*flgJ*, flagellar rod assembly protein	−4.19	6.49
*EAMY_1502*	*fliO*, flagellar biogenesis protein	−4.13	5.93
*EAMY_1512*	*fliE*, flagellar hook-basal body protein	−3.67	5.80
*EAMY_1501*	*fliP*, flagellar biosynthetic protein	−3.54	6.15
*EAMY_1452*	*flgA*, flagellar basal body P-ring biosynthesis protein	−3.3	6.32
*EAMY_1500*	*fliQ*, flagellar biosynthetic protein	−2.81	4.63
*EAMY_2139*	*fliA*, RNA polymerase sigma factor	−2.41	6.33
*EAMY_2138*	*fliZ*, flagellar regulatory protein	−2.15	5.81
*EAMY_2143*	*fliS*, flagellin-specific chaperone	−2.08	6.03
*EAMY_2144*	*fliT*, flagellar export chaperone	−1.89	5.71
*EAMY_2142*	*fliD*, flagellar capping protein	−1.75	7.72
*EAMY_2682*	*fliR*, flagellar biosynthetic protein	−1.54	2.85
*EAMY_1499*	*fliR*, flagellar biosynthetic protein	−1.5	5.59
*EAMY_1463*	*flgL*, flagellar hook-associated protein	−1.28	6.56
*EAMY_1462*	*flgK*, flagellar hook-associated protein	−1.25	7.56
*EAMY_2660*	*flgJ*, flagellar rod assembly protein	−1.00	2.41
*EAMY_2662*	*flgH3*, Flagellar L-ring protein	−1.07	1.70
*EAMY_2689*	*fliK3*, flagellar hook-length control protein	−1.06	3.62

^a^Differentially expressed genes (DEGs) between the (p) ppGpp^0^ mutant and WT at 3 h with |log_2_FC| value ≥ 1 and an adjusted *p* value < 0.05. WT: wild type. FC: fold change. FC values below 0 mean that the gene has lower expression in the (p) ppGpp^0^ mutant than in WT.

^b^log_2_FC valus was calculated by the log based 2 value of (p) ppGpp^0^ / WT at 3 h

^c^The average of log_2_CPM was calculated. CPM: count per million reads

### Differential regulation of amino acid and peptide biosynthesis genes by (p) ppGpp

#### Negative regulation of amino acid biosynthesis by (p) ppGpp

It has been demonstrated that (p) ppGpp and DksA directly activate amino acid biosynthesis under nutrient limited conditions [3, 36]. In contrast, we found that among the 127 DEGs related to amino acid metabolism, 98 (77.2%) were up-regulated in the (p) ppGpp^0^ mutant (Additional file 3**:** Fig. S2a; Table 4), indicating (p) ppGpp negatively regulates genes involved in amino acid biosynthesis. First, the *metREFBKALJ* operon genes for methionine biosynthesis exhibited very high expression in the (p) ppGpp^0^ mutant. Among them, *metR*, encoding a transcriptional regulator, and *metAFE*, which are regulated by *metR* in *E. coli* [37], all showed high positive fold change (log_2_FC = from 3.4 to 5.47). When compared WT at 6 and 3 hpi, methionine biosynthesis genes *metABEFKN* were also up-regulated in WT at 6 h (Table 5), suggesting that methionine might be synthesized in WT at 6 h, whereas (p) ppGpp acts rapidly and robustly to suppress methionine biosynthesis in WT at 3 h. Since methionine plays an important role in translation initiation, it is reasonable for bacteria to synthesize methionine under stress conditions.

**Table 4:** List of differentially expressed genes^a^ associated with amino acid biosynthesis and degradation for the (p) ppGpp^0^ mutant versus WT at 3 h

Accession	Gene Description	Log_**2**_FC^**b**^	Mean^**c**^
**Histidine**			
*EAMY_1255*	*hutH*, histidine ammonia-lyase	6.97	8.62
*EAMY_1254*	*hutU*, urocanate hydratase	5.8	8.85
*EAMY_1259*	*hutI*, imidazolonepropionase	4.93	4.93
*EAMY_1258*	*hutF*, formiminoglutamate deiminase	4.75	6.60
*EAMY_1260*	*hutG*, N-formylglutamate amidohydrolase	3.2	6.31
*EAMY_1256*	*hutC*, transcriptional regulator	1.09	6.71
**Methionine**			
*EAMY_0207*	*metR*, transcriptional regulator	5.47	9.21
*EAMY_0208*	*metE*, methionine synthase II	4.86	13.30
*EAMY_0141*	*metF*, 5,10-methylenetetrahydrofolate reductase	4.71	10.82
*EAMY_0138*	*metB*, cystathionine gamma-synthase	4.56	10.43
*EAMY_0603*	*metK*, S-adenosylmethionine synthetase	4.32	11.75
*EAMY_3342*	*metA*, homoserine transsuccinylase	3.4	9.95
*EAMY_0139*	*metL*, bifunctional aspartokinase	3.31	10.48
*EAMY_0137*	*metJ*, transcriptional regulator of met regulon	2.41	8.53
**Arginine**			
*EAMY_1553*	*argC*, acetylglutamate semialdehyde dehydrogenase	2.41	11.13
*EAMY_0144*	*argB*, acetylglutamate kinase	2.17	10.74
*EAMY_0297*	*argR*, arginine repressor	1.93	8.35
*EAMY_0146*	*argH*, argininosuccinate lyase	1.88	10.84
*EAMY_0145*	*argG*, argininosuccinate synthase	1.83	11.69
*EAMY_3415*	*argD*, 4-aminobutyrate aminotransferase	1.83	10.43
*EAMY_2082*	*argS*, arginyl-tRNA synthetase	1.37	8.58
*EAMY_1631*	*astB*, succinylarginine dihydrolase	−3.05	6.66
*EAMY_1630*	*astD*, NAD-dependent aldehyde dehydrogenase	−1.98	6.45
*EAMY_1628*	*astC*, succinylornithine transaminase	−1.88	7.19
*EAMY_1629*	*astA*, arginine N-succinyltransferase	−1.60	6.15
**Tryptophan**			
*EAMY_1915*	*trpE*, anthranilate synthase component I	3.67	11.22
*EAMY_1916*	*trpG*, anthranilate synthase component II	2.73	8.92
*EAMY_1917*	*trpD*, anthranilate phosphoribosyltransferase	2.4	9.21
*EAMY_1918*	*trpC*, indole-3-glycerol phosphate synthase	1.82	9.45
*EAMY_1919*	*trpB*, tryptophan synthase beta chain	1.49	9.28
*EAMY_3425*	*trpS*, tryptophanyl-tRNA synthetase	1.31	8.67
**Isoleucine**			
*EAMY_0158*	*ilvG*, acetolactate synthase isozyme III large subunit	3.60	10.64
*EAMY_0159*	*ilvM*, acetolactate synthase isozyme II small subunit	2.69	8.25
*EAMY_0160*	*ilvE*, branched-chain amino acidaminotransferase	2.17	9.33
*EAMY_0161*	*ilvD*, dihydroxy-acid dehydratase	1.21	9.57
*EAMY_0163*	*ilvY*, transcriptional regulator	1.12	5.24

^a^Differentially expressed genes (DEGs) between the (p) ppGpp^0^ mutant and WT at 3 h with |log_2_FC| value ≥ 1 and an adjusted *p* value < 0.05. WT: wild type. FC: fold change. FC values below 0 mean that the gene has lower expression in the (p) ppGpp^0^ mutant than in WT, and vice versa

^b^log_2_FC valus was calculated by the log based 2 value of (p) ppGpp^0^ / WT at 3 h

^c^The average of log_2_CPM was calculated. CPM: count per million reads

**Table 5:** List of differentially expressed genes^a^ associated with amino acid biosynthesis and degradation for WT at 6 h versus WT at 3 h

Accession	Gene description	Log_**2**_FC^**b**^	Mean^**c**^
**Methionine**			
*EAMY_0208*	*metE*, methionine synthase II	2.5	10.93
*EAMY_0141*	*metF*, 5,10-methylenetetrahydrofolate reductase	1.92	8.30
*EAMY_0603*	*metK*, S-adenosylmethionine synthetase	1.52	9.40
*EAMY_3342*	*metA*, homoserine transsuccinylase	1.36	8.37
*EAMY_2728*	*metN*, methionine ABC transporter ATP-binding protein	1.15	7.49
*EAMY_0138*	*metB*, cystathionine gamma-synthase	1.04	7.60
**Arginine**			
*EAMY_1630*	*astD*, NAD-dependent aldehyde dehydrogenase	−1.32	6.62
*EAMY_1631*	*astB*, succinylarginine dihydrolase	−1.27	7.00
*EAMY_1628*	*astC*, succinylornithine transaminase	−1.22	7.40
*EAMY_1629*	*astA*, arginine N-succinyltransferase	−1.18	6.31

^a^Differentially expressed genes (DEGs) between the WT at 6 h and at 3 h with |log_2_FC| value ≥ 1 and an adjusted *p* value < 0.05. WT: wild type. FC: fold change. FC values over 0 mean that the gene has higher expression in WT at 6 h than at 3 h. and vice versa

^b^log_2_FC valus was calculated by the log based 2 value of (p) ppGpp^0^ / WT at 3 h

^c^The average of log_2_CPM was calculated. CPM: count per million reads

Second, the *argBCDGHR* operon genes in the arginine biosynthesis pathway [38] were up-regulated in the (p) ppGpp^0^ mutant (1.83 ≤ log_2_FC ≤ 2.41). Consistent with this result, the *astABCD* operon genes, which have been reported for degrading arginine to glutamine [39], were down-regulated (− 1.6 ≤ |log_2_FC| ≤ − 3.05) in the (p) ppGpp^0^ mutant and in WT at 6 h (− 1.18 ≤ |log_2_FC| ≤ − 1.32) as well (Tables 4 and 5). Furthermore, *trpBCDEGS* involved in tryptophan biosynthesis, *livGMEDY* for isoleucine biosynthesis, and *hutCFGHIU* in histidine utility pathway were all up-regulated in the (p) ppGpp^0^ mutant. Among them, the *trpEG* genes, encoding anthranilate synthase [40], *livGM* encoding acetolactate synthase isozymes at the beginning of isoleucine biosynthesis operon, and *hutHUI* genes all exhibited relatively high fold change (Table 4). The *hutHUI* genes have been reported to participate in the degradation of histidine to glutamate which serves as an important donor of amino acid and for nucleotide biosynthesis [41]. Thus, our results indicated that genes involved in the biosynthesis of methionine, arginine, tryptophan, and isoleucine were all negatively regulated by (p) ppGpp, suggesting that up-regulation of these amino acid biosynthesis-related genes in the (p) ppGpp^0^ mutant might be indirect by (p) ppGpp in *E. amylovora*. This is consistent with previous reports that regulation of amino acid biosynthesis genes might be indirect by (p) ppGpp [6, 42]. Sanchez-Vazquez and colleagues found that the promoter of amino acid biosynthesis genes cannot be activated, which was in contrast with other reports [36], and concluded that it might be due to different culture conditions [6]. Consistent with this observation, Traxler and colleagues reported that amino acid biosynthesis genes couldn’t be induced *en masse* in WT under amino acid starvation condition [12]. It is reasonable to speculate that (p) ppGpp negatively regulated amino acid biosynthesis might also be due to the specific growth condition (HMM) used.

It has been reported that amino acid metabolism might be important for virulence [43–45]. In *E. amylovora*, mutants deficient in arginine, isoleucine/valine, and tryptophan metabolism exhibited reduced virulence [44], and the *argD* mutant of *E. amylovora* not only led to arginine auxotrophy, but also exhibited attenuated or no virulence in apples and pears [45]. A methionine metabolism regulator MetR has been identified as a new virulence regulator [46]. Tryptophan biosynthesis gene *trpD* has been reported for its role in inducing quorum-sensing and T3SS in *Pseudomonas aeruginosa* [47]. Durand and Björk reported that a combination of methionine and arginine restore the virulence of the *tgt* mutant, which lacks tRNA and exhibited reduced virulence gene expression in *Shigella flexneri* [43]. A relatively higher expression of methionine and arginine biosynthesis-related genes and down-regulation of arginine degradation genes in the (p) ppGpp^0^ mutant suggest that increased biosynthesis of arginine and/or methionine may help *E. amylovora* survive.

#### Inverse regulation of amino acid and peptide transporter genes by (p) ppGpp

Similar to amino acid biosynthesis genes, 12 out of the17 DEGs related to amino acid ABC (ATP-binding cassette) transport systems were up-regulated in the (p) ppGpp^0^ mutant. Genes (*metNI, EAMY_0862*, and *artPI*) encoded in methionine and arginine import systems were up-regulated in the (p) ppGpp^0^ mutant (1.31 ≤ log_2_FC ≤ 3.24, 1.33 ≤ log_2_FC ≤ 2.24, respectively; Additional file 3: Fig. S2b and Table 6). In addition, seven genes related to polar amino acid uptake transporter (PAAT) were also up-regulated in the (p) ppGpp^0^ mutant (1.12 ≤ log_2_FC ≤ 5.28), though their specific substrates remain unknown. In contrary to amino acid ABC transport systems, 16 DEGs related to peptide ABC transport systems, including genes in the *dpp* and *opp* operons (*dppABCDEF, oppABCDF)* and three genes (*yliD*, *yliC*, *yejA*) belonging to peptide/opine/nickel uptake transporter (PepT) family, were down-regulated in the (p) ppGpp^0^ mutants (Additional file 3: Fig. S2b and Table 6).

**Table 6:** List of differentially expressed genes^a^ associated with amino acid and peptide transport systems for the (p) ppGpp^0^ mutant versus WT at 3 h

Accession	Gene Description	Log_**2**_FC^**b**^	Mean^**c**^
**amino acid transport system**			
**Methionine**			
*EAMY_2728*	*metN*, methionine ABC transporter ATP-binding protein	3.24	9.03
*EAMY_2729*	*metI*, methionine ABC transport system	1.31	7.53
**Arginine (PAAT)**			
*EAMY_0862*	ABC-type arginine/histidine transport system, permease component	2.24	3.66
*EAMY_1315*	*artP*, arginine ABC transport system	1.67	9.20
*EAMY_1314*	*artI*, arginine ABC transport system	1.33	9.86
**Other polar amino acid uptake transport system (PAAT)**			
*EAMY_0860*	ABC transporter substrate-binding protein	5.28	4.96
*EAMY_0266*	*yhdW*, ABC transporter substrate-binding protein	3.47	6.70
*EAMY_0861*	polar amino acid ABC transporter permease	3.36	4.07
*EAMY_0863*	ABC transporter ATP-binding protein	2.52	4.44
*EAMY_0263*	*yhdZ*, ABC-type polar amino acid transport system	1.42	4.52
*EAMY_0264*	*yhdY*, ABC-type amino acid transport system	1.27	4.15
*EAMY_0265*	*yhdX*, ABC-type amino acid transport system	1.12	4.63
**peptide transport system**			
***Opp***			
*EAMY_1936*	*oppB*, ABC transporter permease componenet	−2.65	5.91
*EAMY_1935*	*oppC*, ABC transporter permease componenet	−2.43	5.27
*EAMY_1937*	*oppA*, ABC transporter periplasmic component	−2.29	9.25
*EAMY_1934*	*oppD*, ABC transporter ATPase component	−2.22	5.84
*EAMY_1933*	*oppF*, ABC-type oligopeptide transport system	−1.63	6.52
***Dpp***			
*EAMY_3609*	*dppF*, ABC transporter ATP-binding protein	−2.43	5.77
*EAMY_3611*	*dppC*, transporter	−2.23	5.73
*EAMY_3610*	*dppD*, ABC transporter ATP-binding protein	−2.17	6.15
*EAMY_3613*	*dppA*, ABC transporter periplasmic component	−2.04	9.75
*EAMY_3612*	*dppB*, ABC transporter	−1.94	6.78
**Peptide/Opine/Nickel Uptake Transporter (PepT) Family**			
*EAMY_1292*	*yliD*, ABC-type dipeptide/oligopeptide/nickel transport system	−1.55	6.42
*EAMY_1291*	*yliC*, ABC-type dipeptide/oligopeptide/nickel transport system	−1.41	6.66
*EAMY_2311*	*yejA*, ABC-type oligopeptide transport system	−1.20	6.22

^a^Differentially expressed genes (DEGs) between the (p) ppGpp^0^ mutant and WT at 3 h with |log_2_FC| value ≥ 1 and an adjusted *p* value < 0.05. WT: wild type. FC: fold change. FC values below 0 mean that the gene has lower expression in the (p) ppGpp^0^ mutant than in WT, and vice versa

^b^log_2_FC valus was calculated by the log based 2 value of (p) ppGpp^0^ / WT at 3 h

^c^The average of log_2_CPM was calculated. CPM: count per million reads

Small peptides can be used as carbon and nitrogen sources in bacteria, like *E. coli* and *Salmonella* sp. [48, 49]. Both Opp and Dpp have been reported for importing dipeptides and tripeptides, as well as uptake of essential amino acids in *Streptococcus pyogenes* [50, 51]. The Opp system also recycles cell-wall peptide and senses environment [49, 52]. Kim and colleagues suggested that peptide transporters provide peptides containing essential amino acids for both survival and infection in *Salmonella* [53]. Previous study showed that both Opp and Dpp are hijacked for importing antibiotics, but are dispensable for virulence in *E. amylovora* [54]. Taken together, these results suggested that (p) ppGpp positively regulates peptide uptake systems in WT, but negatively regulates genes involved in amino acid uptake systems and amino acid biosynthesis in the HMM environment.

### Negative regulation of genes contributing to survival by (p) ppGpp

#### Translation

Inhibition of (p) ppGpp in translation by repressing the synthesis of tRNA, rRNA and ribosome has been well documented [1, 26, 55]. The slow growth of the (p) ppGpp^0^ mutant [17] might be related to negative regulation of (p) ppGpp in ribosomal proteins as reported previously [56]. Consistently, 98 of 106 genes (92.5%) related to translation were up-regulated in the (p) ppGpp^0^ mutant. Among them, 33 genes (*rps*, *rpm*, and *rpl*) associated with ribosomal subunits were up-regulated in the (p) ppGpp mutant (1.15 ≤ log_2_FC ≤ 2.86; Additional file 3: Fig. S2c and Table 7), indicating that (p) ppGpp negatively mediates ribosomal protein biosynthesis. Lemke and colleagues found that r-protein promoter activities decreased in WT after SHX treatment, suggesting a direct negative regulation by ppGpp and DksA [55]. Besides ribosomal protein genes, *infA* and *tufA*, encoding a translation initiation factor and a translation elongation factor, respectively, were also up-regulated in the (p) ppGpp^0^ mutant (log_2_FC = 1.52 & 2.24), indicating that (p) ppGpp negatively regulates translation through down-regulating initiation and elongation factors. Srivatsan and Wang reported that (p) ppGpp inhibits and interferes the functions of the initiation factor IF2 and the elongation factors EF-Tu and EF-G in *E coli*. In addition, (p) ppGpp binds to IF2 and EF-G to inhibit translation when competing with GDP and GTP [26]. Overall, (p) ppGpp might control translation capacity in the cell to prevent the depletion of cell resources under stress conditions.

**Table 7:** List of differentially expressed genes^a^ associated with translation for the (p) ppGpp^0^ mutant versus WT at 3 h

Accession	Gene Description	Log_**2**_FC^**b**^	Mean^**c**^
**Translation initiation**			
*EAMY_1327*	*infA*, translation initiation factor IF-1	1.52	9.39
***Translation elongation***			
*EAMY_0232*	*tufA*, translation elongation factors	2.24	12.20
**ribosomal protein synthesis**			
***rpl***			
*EAMY_0303*	*rplM*, ribosomal protein L13	2.70	12.08
*EAMY_2320*	*rplY*, ribosomal protein L25	2.65	11.32
*EAMY_3376*	*rplN*, ribosomal protein L14	1.93	11.83
*EAMY_3375*	*rplX*, ribosomal protein L24	1.93	11.86
*EAMY_3374*	*rplE*, ribosomal protein L5	1.90	12.34
*EAMY_0236*	*rplA*, ribosomal protein L1	1.81	10.97
*EAMY_3371*	*rplF*, ribosomal protein L9	1.75	11.37
*EAMY_0235*	*rplK*, ribosomal protein L11	1.68	10.54
*EAMY_3370*	*rplR*, ribosomal protein L18	1.60	10.61
*EAMY_3367*	*rplO*, ribosomal protein L15	1.54	11.59
*EAMY_3142*	*rplI*, ribosomal protein L9	1.40	10.49
*EAMY_0332*	*rplU*, ribosomal protein L21	1.36	10.76
*EAMY_3360*	*rplQ*, ribosomal protein L17	1.27	10.39
*EAMY_3386*	*rplC*, ribosomal protein L3	1.17	11.07
*EAMY_3385*	*rplD*, ribosomal protein L4	1.15	10.38
***rpm***			
*EAMY_0136*	*rpmE*, ribosomal protein L31	2.64	9.97
*EAMY_0078*	*rpmG*, ribosomal protein L33	2.43	10.07
*EAMY_3368*	*rpmD*, ribosomal protein L30	1.8	10.52
*EAMY_0077*	*rpmB*, ribosomal protein L28	1.76	10.47
*EAMY_0333*	*rpmA*, ribosomal protein L27	1.64	11.06
***rps***			
*EAMY_0304*	*rpsI*, ribosomal protein S9	2.86	11.55
*EAMY_0352*	*rpsO*, ribosomal protein S15	2.51	10.25
*EAMY_0417*	*rpsU*, ribosomal protein S21	2.12	10.15
*EAMY_2940*	*rpsT*, ribosomal protein S20	1.84	10.18
*EAMY_2760*	*rpsB*, ribosomal protein S2	1.82	11.96
*EAMY_3373*	*rpsN*, ribosomal protein S14	1.76	11.7
*EAMY_3372*	*rpsH*, ribosomal protein S8	1.72	11.23
*EAMY_3369*	*rpsE*, ribosomal protein S5	1.57	11.12
*EAMY_3143*	*rpsR*, ribosomal protein S18	1.30	10.04
*EAMY_3390*	*rpsG*, ribosomal protein S7	1.26	10.84
*EAMY_0816*	*rpsP*, ribosomal protein S16	1.22	9.43
*EAMY_3145*	*rpsF*, ribosomal protein S6	1.21	10.8
*EAMY_3387*	*rpsJ*, ribosomal protein S10	1.24	10.35

^a^Differentially expressed genes (DEGs) between the (p) ppGpp^0^ mutant and WT at 3 h with |log_2_FC| value ≥ 1 and an adjusted *p* value < 0.05. WT: wild type. FC: fold change. FC values over 0 mean that the gene has higher expression in the (p) ppGpp^0^ mutant than in WT.

^b^log_2_FC valus was calculated by the log based 2 value of (p) ppGpp^0^ / WT at 3 h

^c^The average of log_2_CPM was calculated. CPM: count per million reads

#### Biosynthesis of purine and pyrimidine

Thirty eight out of 47 DEGs (80.9%) related to nucleotide metabolism were up-regulated in the (p) ppGpp^0^ mutant. Among them, 14 and 12 DEGs are related to purine and pyrimidine biosynthesis, respectively (Additional file 3: Fig. S2d and Table 8). The *purCDHIMNTU* operon genes (1.68 ≤ log_2_FC ≤ 1.86) are involved in synthesizing inosine monophosphate (IMP), a nucleotide monophosphate for generating AMP and GMP from 5-phosphoribosyl diphosphate (PRPP) in *E. coli* [57]. The *deoD* and *gpt* genes (log_2_FC = 2.41 & 1.97, respectively) were involved in purine salvage pathway for synthesizing IMP from hypoxanthine [57]. Moreover, two GMP synthesis genes, *guaA* and *guaB*, were also up-regulated in the (p) ppGpp^0^ mutant (log_2_FC = 1.74 and 3.02, respectively), which supported a previous report of an uncontrollable increase of GTP level (~ 10 mM or higher) in the (p) ppGpp^0^ mutant [58]. In consistent with our results, (p) ppGpp has been reported to inhibit enzymes that initiate ATP and GTP biosynthesis [1, 59]. Furthermore, several genes in both UMP de novo biosynthesis pathway (*carAB* and *pyrBFI*), UMP salvage pathway (*udp* and *udk*), and CMP biosynthesis-related genes (*pyrG* and *cmK*) were up-regulated in the (p) ppGpp^0^ mutant (Additional file 3: Fig. S2d and Table 8). UMP is the precursor of CTP biosynthesis, and PyrG/CTP synthase is an importance enzyme for the conversion of UMP to CMP [60]. Overall, these results indicate that (p) ppGpp negative controls purine and pyrimidine biosynthesis pathways [27, 58].

**Table 8:** List of differentially expressed genes^a^ associated with nucleitide metabolism for the (p) ppGpp^0^ mutant versus WT at 3 h

Accession	Gene Description	Log_**2**_FC	Mean^**b**^
**Purine**			
GMP			
*EAMY_2568*	*guaB*, inosine-5′-monophosphate dehydrogenase	2.94	10.85
*EAMY_2567*	*guaA*, GMP synthase	1.66	9.06
*EAMY_2859*	*guaC*, GMP reductase	1.55	8.44
IMP			
*EAMY_2052*	*purT*, phosphoribosylglycinamide formyltransferase II	1.79	9.79
*EAMY_2542*	*purM*, phosphoribosylformylglycinamidine cyclo-ligase	1.74	10.49
*EAMY_2610*	*purl*, FGAM synthase	1.5	10.89
*EAMY_2529*	*purC*, SAICAR synthase	1.4	9.8
*EAMY_0262*	*purH*, bifunctional purine biosynthesis protein	1.28	9.07
*EAMY_1965*	*purU*, formyltetrahydrofolate deformylase	1.16	8.47
*EAMY_2543*	*purN*, phosphoribosylglycinamide formyltransferase	1.02	8.57
*EAMY_0261*	*purD*, phosphoribosylamine-glycine ligase	1	9.04
*EAMY_2978*	*deoD*, uridine phosphorylase	2.34	9.11
*EAMY_0884*	*gpt*, xanthine phosphoribosyltransferase	1.89	7.3
*EAMY_0864*	*pucG*, serine-pyruvate aminotransferase	1.59	5.14
**Pyrimidine**			
UMP			
*EAMY_1900*	*pyrF*, orotidine-5′-phosphate decarboxylase	2.16	7.42
*EAMY_2932*	*carA*, carbamoyl-phosphate synthase small subunit	1.93	11.18
*EAMY_2283*	*cdd*, cytidine deaminase	1.88	6.95
*EAMY_0366*	*pyrB*, aspartate carbamoyltransferase	1.83	8.42
*EAMY_2931*	*carB*, carbamoyl-phosphate synthase large chain	1.25	11.53
*EAMY_0074*	*dut*, deoxyuridine 5′-triphosphate nucleotidohydrolase	1.53	7.54
*EAMY_0210*	*udp*, uridine phosphorylase	1.47	7.34
*EAMY_0365*	*pyrI*, aspartate carbamoyltransferase	1.27	7.49
*EAMY_2257*	*udk*, uridine kinase	1.04	7.62
CMP			
*EAMY_0737*	*pyrG*, CTP synthase	2.12	10.37
*EAMY_1346*	*cmK*, cytidylate kinase	1.06	7.98
TMP			
*EAMY_2980*	*deoA*, thymidine phosphorylase	1.63	7.04

^a^Differentially expressed genes (DEGs) between the (p) ppGpp^0^ mutant and WT at 3 h with |log_2_FC| value ≥ 1 and an adjusted *p* value < 0.05. WT: wild type. FC: fold change. FC values over 0 mean that the gene has higher expression in the (p) ppGpp^0^ mutant than in 1valus was calculated by the log based 2 value of (p) ppGpp^0^ / WT at 3 h

^c^The average of log_2_CPM was calculated. CPM: count per million reads

#### DNA replication/recombination/repair

Thirty out of 37 DEGs (81.1%) related to replication/recombination/repair were up-regulated in the (p) ppGpp^0^ mutant (Additional file 2: Table S2). Among them, 11 genes were involved in DNA-inducible SOS function (Additional file 3: Fig. S2e and Table 9). Two SOS response-associated genes (*recAN*) and an inhibitor of SOS response gene *lexA* were all highly expressed in the (p) ppGpp^0^ mutant (log_2_FC = 1.97, 2.96 and 6.95; respectively). The *recA* gene activates the *recN* gene, and helps co-ordinate the recombination of DNA double strand breaks [61]. Whereas LexA could self-cleavage in the present of RecA [62]. Under severe DNA damage, expression of the *recA-lexA* genes could result in an apoptosis-like death as an extreme SOS response in *E coli* [63]. In addition, several SOS response-associated genes, including *dinP*, *ruvA* and *ruvB*, which have been reported being repressed by (p) ppGpp under amino acid starvation [64], were also up-regulated in the (p) ppGpp^0^ mutant (log_2_FC = 2.15, 1.66, 1.18, respectively). Kim and colleagues found that overexpression of *dinB/dinP* resulted in enhancing mutagenesis in *E. coli* [65]. Therefore, expression of large number of DNA repair and SOS inducible genes indicates that DNA damage or mismatch may commonly occur in the (p) ppGpp^0^ mutant, which eventually leads to cell death as reported previously [16].

**Table 9:** List of differentially expressed genes^a^ associated with DNA repair/replication for the (p) ppGpp^0^ mutant versus WT at 3 h

Accession	Gene Description	Log_**2**_FC	Mean^**b**^
**DNA-repair: SOS response**			
*EAMY_2641*	*recN*, DNA repair protein	2.96	9.84
*EAMY_3327*	*lexA*, SOS-response transcriptional repressors	2.95	10.27
*EAMY_3296*	*ssb*, single-stranded DNA-binding protein	2.22	9.95
*EAMY_0882*	*dinP*, DNA polymerase IV	2.15	6.96
*EAMY_0805*	*recA*, recombinase A	1.97	11.18
*EAMY_2064*	*ruvA*, Holliday junction ATP-dependent DNA helicase	1.66	6.84
*EAMY_1211*	*uvrB*, excinuclease UvrABC subunit B	1.56	8.45
*EAMY_1251*	*dinG*, ATP-dependent helicase	1.23	7.05
*EAMY_2063*	*ruvB*, Holliday junction ATP-dependent DNA helicase	1.18	7.44
*EAMY_0194*	*uvrD*, DNA helicase II	1.13	8.55
*EAMY_3297*	*uvrA*, excinuclease ATPase subunit	1.01	9.29
**DNA replication**			
*EAMY_3296*	*ssb*, single-stranded DNA-binding protein	2.22	9.95
*EAMY_2345*	*gyrA*, DNA gyrase A subunit	1.64	10.46
*EAMY_0725*	*exo*, 5′-3′ exonuclease	1.47	6.31
*EAMY_0844*	*dnaQ*, DNA polymerase III epsilon subunit	1.36	6.88
*EAMY_3144*	*priB*, primosomal replication protein N	1.24	10.24
*EAMY_1122*	*holA*, DNA polymerase III delta subunit	−1.21	6.86

^a^Differentially expressed genes (DEGs) between the (p) ppGpp^0^ mutant and WT at 3 h with |log_2_FC| value ≥ 1 and an adjusted *p* value < 0.05. WT: wild type. FC: fold change. FC values below 0 mean that the gene has lower expression in the (p) ppGpp^0^ mutant than in WT, and vice versa

^b^log_2_FC valus was calculated by the log based 2 value of (p) ppGpp^0^ / WT at 3 h

^c^The average of log_2_CPM was calculated. CPM: count per million reads

It has been reported that DNA replication was inhibited by (p) ppGpp [66]. Consistently, five genes related to DNA replication were up-regulated in the (p) ppGpp^0^ mutant (Additional file 3: Fig. S2e and Table 9). The *ssb* gene, encoding a single strand DNA-binding protein, is essential for DNA replication, recombination and repair [67], and is also involved in SOS system [68]. Another gene encoding a DNA polymerase III subunit epsilon processes a proofreading function of polymerase III holoenzyme [69]. It has been reported that replication forks arrested under amino acid starvation conditions, especially at the time of replication initiation [70]. It is possible that rapid and reversible replication arrest might help bacteria stabilize genome DNA during starvation.

#### Fatty acid/lipid metabolism and cell cycle

Sixteen out of 23 DEGs involved in lipid metabolism were up-regulated in the (p) ppGpp^0^ mutant (Additional file 3: Fig. S2f and Table 10), including the *fabBZ* genes, which are involved in unsaturated fatty acid biosynthesis. A fatty acid degradation gene *fadA*, on the other hand, was down-regulated in the (p) ppGpp^0^ mutant (|log_2_FC| = − 1.22), indicating that (p) ppGpp negatively regulates fatty acid biosynthesis genes in *E. amylovora*. It has been reported that both *fabB* and *fadA* are under control of a dual transcriptional regulator *fadR*. During fatty acid starvation, *fadR* represses *fadA* operon to prevent fatty acid degradation [71] and activates *fabB* to enhance fatty acid synthesis [72]. In *E. coli*, (p) ppGpp and DksA inhibited *fadH* expression directly or indirectly through *fadR* to down-regulate fatty acid biosynthesis [73].

**Table 10:** List of differentially expressed genes^a^ associated with lipid metabolism/cell cycle for the (p) ppGpp^0^ mutant versus WT at 3 h

Accession	Gene Description	Log_**2**_FC^**b**^	Mean^**c**^
**Lipid metabolism**			
**Fatty acid biosynthesis**			
*EAMY_2423*	*fabB*, 3-oxoacyl-(acyl-carrier-protein) synthase	2.38	11.6
*EAMY_2408*	*accD*, acetyl-CoA carboxylase beta subunit	1.86	9.97
*EAMY_2748*	*fabZ*, 3-hydroxymyristoyl/3-hydroxydecanoyl-(acyl carrier protein) dehydratases	1.46	8.39
*EAMY_0948*	*yajB*, acyl carrier protein phosphodiesterase	1.2	5.16
*EAMY_1242*	*cfa*, cyclopropane fatty acid synthase	−1.7	6.96
**Fatty acid degradation**			
*EAMY_2827*	*vraB*, 3-ketoacyl-CoA thiolase	−1.72	4.77
*EAMY_0222*	*fadA*, acetyl-CoA acetyltransferase	−1.22	10.1
**Cell division**			
**chromosom partition**			
*EAMY_1925*	intracellular septation protein A	1.8	7.37
*EAMY_1357*	*mukF*, chromosome partition protein	1.75	8.20
*EAMY_2278*	*mrp*, ATPases involved in chromosome partitioning	1.36	8.77
*EAMY_1358*	*mukE*, chromosome partition protein	1.27	7.22
**cell division**			
*EAMY_1387*	*sulA*, cell division inhibitor	2.19	8.22
*EAMY_0129*	*zapB*, cell division protein	1.2	8.58
*EAMY_2482*	*zipA*, cell division protein	1.12	9.29

^a^Differentially expressed genes (DEGs) between the (p) ppGpp^0^ mutant and WT at 3 h with |log_2_FC| value ≥ 1 and an adjusted *p* value < 0.05. FC: fold change. WT: wild type. WT: wild type. FC: fold change. FC values below 0 mean that the gene has lower expression in the (p) ppGpp^0^ mutant than in WT, and vice versa

^b^log_2_FC valus was calculated by the log based 2 value of (p) ppGpp^0^ / WT at 3 h

^c^The average of log_2_CPM was calculated. CPM: count per million reads

In addition, eight out of nine DEGs involved in cell cycle were up-regulated in the (p) ppGpp^0^ mutant (Additional file 3: Fig. S2f and Table 10). The *mukEF* genes are involved in chromosome condensation and segregation [74]. Ferullo and Lovett showed that chromosome segregation was arrested by (p) ppGpp in *E. coli* after SHX treatment [75]. Moreover, genes related to cell division (*sulA, zapB, zipA*) were also up-regulated in the (p) ppGpp^0^ mutant (Additional file 3: Fig. S2f and Table 10). The *sulA* gene, encoding a cell division inhibitor, and the *zapB* and *zipA* genes are all essential for cell division [76, 77]. Accumulation of SulA protein causes rapid arrest of cell division, resulting in long and non-separate filament [76]. Indeed, the (p) ppGpp^0^ mutant exhibited longer cells in both *E amylovora* [17] and *P. syringae* [16]. Traxler and colleagues showed that the (p) ppGpp^0^ mutant produced an average of around 50% more biomass than that of the WT under isoleucine limited condition [12]. Taken together, DNA replication, biosynthesis of nucleotide metabolism, cell wall, fatty acid, as well as cell division all contribute to biomass [12]. The lack of (p) ppGpp caused abnormal up-regulation of DNA replication, biosynthesis of nucleotides, cell wall, fatty acid, as well as cell division genes, which may further deplete cell resources, eventually leading to cell death.

## Conclusions

**Fig. 7:**
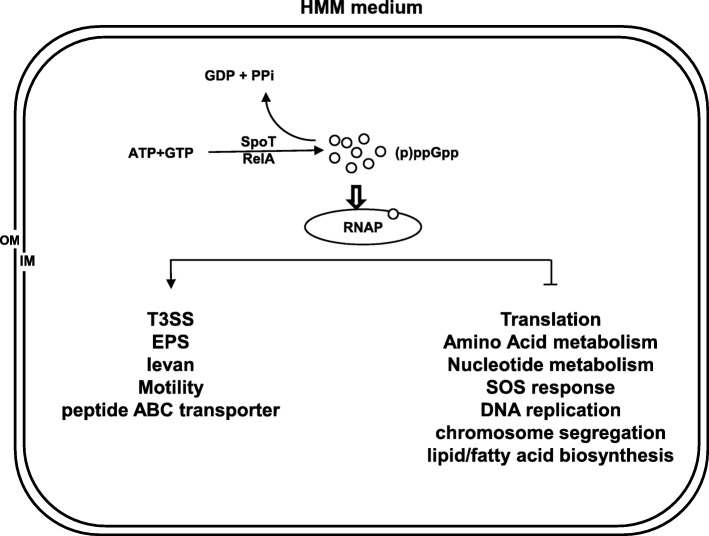
Proposed working model for (p) ppGpp regulation in *E. amylovoran*. RNAP: RNA polymerase; Symbols: orange spots: (p) ppGpp; blue oval: RNAP; downwards arrow: positive effect; box drawings light up and horizontal: negative effect

Based on our current as well as previous reported results [17], a simple working model was proposed (Fig. 7). When *E. amylovora* tries to colonize plant and starts its infection process, perturbations, such as limited nutrients, acidity, or oxidative stress, activate the RelA/SpoT system and promote (p) ppGpp production. In HMM medium, the (p) ppGpp triggers the expression of T3SS, motility and peptide ABC transporter genes. Simultaneously, genes for biosynthesis of amino acid, and nucleotide, fatty acid, lipid, SOS system, DNA replication, chromosome segregation, as well as translation are suppressed by (p) ppGpp. In this environment, (p) ppGpp redistributes cell resources to virulence gene expression, and at the same time maintains the balance between survival by its quick reversal of the stringent response.

## Methods

### Bacterial strains and growth conditions

The *E. amylovora* WT strain Ea1189 and the *relA/spoT* double mutant strain, i. e. the (p) ppGpp^0^ mutant [17], were routinely grown in Luria-Bertani (LB) broth. The *hrp*-inducing minimal medium (HMM) (1 g (NH_4_)_2_SO_4_, 0.246 g MgCl_2_ • 6H_2_O, 0.1 g NaCl, 8.708 g K_2_HPO_4_, 6.804 g KH_2_PO_4_]/Liter) supplemented with 10 mM galactose as carbon source, was used for T3SS gene expression and RNA-seq [17, 20]. Antibiotics were used at the following concentrations when appropriate: 50 μg/mL kanamycin (Km) and 25 μg/mL chloramphenicol (Cm). Primers used in this study were listed in Table S3 (Additional file 4).

### RNA isolation and Illumina sequencing

Bacteria strains cultured overnight in LB broth at 28 °C with appropriate antibiotics were collected by centrifugation at 4000 rpm and washed three times in HMM before being inoculated into 5 mL of HMM at OD_600_ of 0.2 [20]. After 3 and 6 h inoculation at 18 °C with shaking at 250 rpm, 4 ml of RNA protected reagent (Qiagen, Hilden, Germany) was added to 2 ml of bacteria culture, mixed by vortexing, and incubated for 5 min at room temperature to prevent RNA degradation. RNA was extracted by RNeasy® minikit (Qiagen, Hilten, Germany) following the manufacturer’s instructions, and DNase I treatment was performed with a Turbo DNA-free kit (ambion, Austin, TX). The quantity and quality of RNA samples were assessed using NanoDrop ND-100 spectrophotometer (NanoDrop Technologies, Wilmington, DE, USA) and/or using Agilent RNA 6000 Nano Chip Bioanalyzer (Agilent, Santa Clara, CA, USA). Three biological samples each for WT-3 h, WT-6 h, and (p) ppGpp^0^-3 h were then sent to the Keck Center at the University of Illinois at Urbana-Champaign for library constructions and Illumina HiSeq 2500 (Illumina, San Diego, CA, USA) sequencing. A total of nine stranded libraries were constructed using TruSeq Stranded RNA Sample Prep kit following the manufacturer’s instructions (Illumina, San Diego, CA, USA).

### Transcriptomic data profiling and differentially expressed gene detection

The RNA-seq reads were aligned to the reference coding sequences (CDSs) of *E. amylovora* strain CFBF1430 [78], using the default parameters of the Burrows-Wheeler Aligner (version 0.12.7) [79] (http://bio-bwa.sourceforge.net/). Samtools and bedtools were performed for getting the read counts per CDS. Normalized log2-based count per million values (log_2_CPM) were calculated after TMM (trimmed mean of M values) normalization in the edgeR package [80, 81]. To examine gene expression dynamics among all the samples (WT-6 h, WT-3 h, (p) ppGpp^0^-3 h), a principle component analysis (PCA) was conducted by using prcomp in R. Differentially expressed gene (DEGs) between comparisons ((p) ppGpp^0^/WT-3 h and WT-6 h/WT-3 h) were detected in edgeR package [80, 81] and screened by a statistics filter (*P* < 0.05, |log_2_FC| > 1). For functionally categorization of DEGs using COGs, protein sequence of all coding genes were downloaded from NCBI (https://www.ncbi.nlm.nih.gov/). The two FASTA protein files were used as input for protein annotation using eggNOG-mapper (http://eggnogdb.embl.de/#/ app/emapper). COG information for DEGs was extracted from eggNOG output file. The RNA-seq data files have been submitted to Gene Expression Omnibus (GEO) at the National Center for Biotechnology Information (NCBI) with an accession number GSE143324 and GSE128088.

### Quantitative reverse transcription real-time polymerase chain reaction (qRT-PCR)

One microgram of total RNA was reversed transcribed to cDNA using Superscript III reverse transcriptase (Invitrogen, Carlsbad, CA, USA) following the manufacturer’s instructions. Power SYBR® Green PCR master mix (Applied Biosystems, Foster City, CA, USA) with appropriate primers (Additional file 3: Table S3) was mixed with one microliter of cDNAs, and qRT-PCR was performed using the StepOnePlus Real-Time PCR system (Applied Biosystems) under the following conditions: 95 °C for 10 min, followed by 40 cycles of 95 °C for 15 s and 60 °C for 1 min. The dissociation curve was measured after the program was completed, and relative gene expression was calculated with the relative quantification (ΔΔCt) method using the *rpoD* gene as an endogenous control. A *P*-value was computed using student t-test to measure the significance associated with each relative quantification value. Variations were statistically significant when *P* < 0.05. The experiment was repeated at least twice.

## Supplementary information

**Additional file 1: Table S1.** List of differentially expressed genes (DEGs) of (p) ppGpp^0^ versus Ea1189.

**Additional file 2: Table S2.** List of DEGs of Ea1189 at 6 h versus at 3 h.

**Additional file 3: Figure S1.** Motility of the wild type Ea1189 and the *∆relA/spoT* mutant on soft tryptone agar plates (3%) at 28 °C and photographs were taken after 48 h. **Fig. S2**. Differentially expressed genes negatively regulated by (p) ppGpp. (a) amino acid biosynthesis and degradation. (b) amino acid and peptide transport systems. (c) translation (d) nucleotide metabolism. (e) DNA repair/replication (f) lipid metabolism/cell cycle. White represents mean of expression level (log_2_CPM), dark blue represents minimal gene expression, and bright red represents maximal gene expression. In the side bar (right), dark green represents lower negative fold change (log_2_FC), and bright red represents the higher positive log_2_FC.

**Additional file 4: Table S3.** Primers for qRT-PCR used in this study.

## Data Availability

The datasets generated during the current study are available in the Gene Expression Omnibus (GEO) at the National Center for Biotechnology Information (NCBI) with an accession number GSE143324 and GSE128088.

## Abbreviations

(p) ppGpp: guanosine tetraphosphate and pentaphosphate
T3SS: Type III secretion system
DEGs: Differentially expressed genes
ABC: ATP-binding cassette
RSH: RelA/SpoT homologue
RNAP: RNA polymerase
SHX: Serine hydroxamate
*hrp*: hypersensitive response and pathogenicity
RpoN: Alternative sigma factor 54
WT: Wild-type
hpi: hour post incubation
PCA: Principal component analysis
CPM: Counts per million reads
COG: Clusters of orthologous groups
HMM: *Hrp*-inducing medium
FC: Fold change
LB: Luria-Bertani
Km: Kanamycin
Cm: Chloramphenicol
CDS: Coding sequence
TMM: Trimmed mean of M values
GEO: Gene expression omnibus
NCBI: National center for biotechnology information
qRT-PCR: quantitative reverse transcription real-time polymerase chain reaction
