# Supplementary material for: The stringent response regulator (p) ppGpp mediates virulence gene expression and survival in Erwinia amylovora
Source: BMC Genomics. 2020 Mar 30;21:261. doi: 10.1186/s12864-020-6699-5 (PMC7106674; doi:10.1186/s12864-020-6699-5)
Supplement: Supplementary file 1 — Additional file 1: Table S1. List of differentially expressed genes (DEGs) of (p) ppGpp0 versus Ea1189. [file 12864_2020_6699_MOESM1_ESM.pdf]

Table S1: Differentially expressed genes from RNA-seq analyses of WT and the *relA*/*spoT* mutant after 3 h incubation in the *hrp*-inducing medium. Differentially expressed genes are grouped into functional categories according to the clusters of orthologous group (COG) database: FC, fold changes

A) Up-regulated genes in the *relA*/*spoT* mutant (adjusted *P*-value < 0.05)

Locus tag	Gene description	log_2FC
**Amino acid transport and metabolism**		
EAMY_1255	*hutH*, histidine ammonia-lyase	6.97
EAMY_1254	*hutU*, urocanate hydratase	5.80
EAMY_0860	ABC transporter substrate-binding protein	5.28
EAMY_0826	*aroF*, phospho-2-dehydro-3-deoxyheptonate aldolase	4.96
EAMY_0208	*metE*, methionine synthase II	4.86
EAMY_0141	*metF*, 5,10-methylenetetrahydrofolate reductase	4.71
EAMY_0138	*metB*, cystathionine gamma-synthase	4.56
EAMY_0995	*glnK*, nitrogen regulatory protein PII	4.12
EAMY_0876	*eif*, translation initiation factor EIF-2B	4.07
EAMY_1915	*trpE*, anthranilate synthase component I	3.67
EAMY_0158	*ilvG*, acetolactate synthase isozyme III large subunit	3.60
EAMY_0266	ABC transporter substrate-binding protein	3.47
EAMY_3342	*metA*, homoserine transsuccinylase	3.40
EAMY_0861	polar amino acid ABC transporter permease	3.36
EAMY_1071	homocysteine *S*-methyltransferase family protein	3.31
EAMY_0139	*metL*, bifunctional aspartokinase	3.31
EAMY_2728	*metN*, methionine ABC transporter ATP-binding protein	3.24
EAMY_0874	*masA*, enolase-phosphatase	3.22
EAMY_1260	*hutG*, *N*-formylglutamate amidohydrolase	3.20
EAMY_0856	gamma-glutamyltranspeptidase	3.17
EAMY_1072	*ophA*, ABC transporter substrate-binding protein	3.14
EAMY_0941	*yxjG*, methionine synthase II	3.13
EAMY_0877	*mtnK*, 5-methylthioribose kinase	3.09
EAMY_0827	*tyrA*, prephenate dehydrogenase	2.94
EAMY_2933	*dapB*, dihydrodipicolinate reductase	2.81
EAMY_1916	*trpG*, anthranilate synthase component II	2.73
EAMY_0863	ABC transporter ATP-binding protein	2.52
EAMY_0875	dioxygenase	2.47
EAMY_1553	*argC*, acetylglutamate semialdehyde dehydrogenase	2.41
EAMY_0137	*metJ*, transcriptional regulator of *met* regulon	2.41
EAMY_1917	*trpD*, anthranilate phosphoribosyltransferase	2.40
EAMY_2930	Putative threonine efflux protein	2.35
EAMY_0862	ABC-type arginine/histidine transport system, permease	2.24

	component	
EAMY_0160	*ilvE*, branched-chain amino acidaminotransferase	2.17
EAMY_0144	*argB*, acetylglutamate kinase	2.17
EAMY_2906	*leuA*, 2-isopropylmalate synthase	2.09
EAMY_0465	*dat*, diaminobutyrate-2-oxoglutarateaminotransferase	2.04
EAMY_1210	ABC-type spermidine/putrescine transport	2.03
EAMY_2932	*carA*, carbamoyl-phosphate synthase small subunit	2.01
EAMY_1147	*mtr*, amino acid permease	1.91
EAMY_0146	*argH*, argininosuccinate lyase	1.88
EAMY_0145	*argG*, argininosuccinate synthase	1.83
EAMY_3415	*argD*, 4-aminobutyrate aminotransferase	1.83
EAMY_1918	*trpC*, indole-3-glycerol phosphate synthase	1.82
EAMY_3339	*lysC*, aspartate kinase	1.77
EAMY_1749	*dcp*, dipeptidyl carboxypeptidase II	1.74
EAMY_2951	*thrA*, homoserine dehydrogenase	1.74
EAMY_2907	*leuB*, 2-isopropylmalate synthase	1.73
EAMY_1139	*asnB*, asparagine synthase	1.69
EAMY_0864	*pucG*, serine-pyruvate aminotransferase	1.68
EAMY_1315	*artP*, arginine ABC transport system	1.67
EAMY_0036	*glnA*, glutamine synthetase	1.67
EAMY_0578	*speC*, ornithine decarboxylase isozyme	1.66
EAMY_2597	*iscS*, cysteine desulfurase	1.63
EAMY_3256	*rhtB*, putative threonine efflux protein	1.52
EAMY_2596	*pepB*, leucyl aminopeptidase	1.50
EAMY_2504	*ansP*, *L*-asparagine permease	1.49
EAMY_1919	*trpB*, tryptophan synthase beta chain	1.49
EAMY_2613	hypothetical protein	1.47
EAMY_3471	*asd*, aspartate-semialdehyde dehydrogenase	1.46
EAMY_0263	*yhdZ*, ABC-type polar amino acid transport system	1.42
EAMY_2604	*glyA*, glycine/serine hydroxymethyltransferase	1.41
EAMY_2606	*glnB*, nitrogen regulatory protein PII	1.39
EAMY_0847	*hyuE*, hydantoin racemase	1.38
EAMY_2975	*serB*, phosphoserine phosphatase	1.36
EAMY_2931	*carB*, carbamoyl-phosphate synthase large chain	1.33
EAMY_0224	*pepQ*, proline dipeptidase	1.33
EAMY_1314	*artI*, arginine ABC transport system	1.33
EAMY_2531	*dapA*, dihydrodipicolinate synthase	1.32
EAMY_3416	*pabA*, glutamine amidotransferase	1.31
EAMY_2729	*metI*, methionine ABC transport system	1.31
EAMY_0636	*pepP*, proline aminopeptidase P II	1.28
EAMY_1712	*ydgR*, tripeptide transporter permease	1.28

EAMY_0264	*yhdY*, ABC-type amino acid transport system	1.27
EAMY_0466	*ddc*, *L*-2,4-diaminobutyrate decarboxylase	1.27
EAMY_0919	*aroL*, shikimate kinase II	1.24
EAMY_2908	*leuC*, aconitase A	1.24
EAMY_0753	*cysD*, sulfate adenylyltransferase subunit II	1.23
EAMY_0161	*ilvD*, dihydroxy-acid dehydratase	1.21
EAMY_0640	*gcvT*, aminomethyltransferase	1.19
EAMY_2429	*aroC*, chorismate synthase	1.17
EAMY_2950	homoserine kinase	1.17
EAMY_0883	*pepD*, aminoacyl-histidine dipeptidase	1.16
EAMY_1073	*ophB*, ABC transporter	1.13
EAMY_0265	*yhdX*, ABC-type amino acid transport system	1.12
EAMY_2727	*yaeD*, histidinol phosphatase	1.12
EAMY_3182	*aspA*, aspartate ammonia-lyase	1.11
EAMY_3303	*tyrB*, aromatic amino acid transaminase	1.11
EAMY_2012	*sdaA*, L-serine dehydratase	1.10
EAMY_3099	*sstT*, sodium/serine symporter	1.08
EAMY_0714	*argA*, acetylglutamate kinase	1.07
EAMY_2412	*usg*, aspartate-semialdehyde dehydrogenase	1.06
EAMY_1603	*prsA*, phosphoribosylpyrophosphate synthetase	1.00
**Carbohydrate transport and metabolism**		
EAMY_3649	ABC transporter ATP-binding protein	4.64
EAMY_3648	ABC transporter permease component	4.06
EAMY_0873	methylthioribulose-1-phosphate dehydratase	3.64
EAMY_3647	ABC transporter substrate-binding protein	3.37
EAMY_3566	*gnl*, gluconolactonase	3.21
EAMY_1738	*ynfM*, major facilitator superfamily transporter	2.96
EAMY_2305	*fruB*, PTS system fructose-specific EIIA component	2.69
EAMY_2103	*otsB*, trehalose-6-phosphate phophatase	2.52
EAMY_2552	major facilitator superfamily transporter	2.42
EAMY_2303	*fruA*, PTS system fructose-specific EIIC component	2.32
EAMY_2979	*deoB*, phosphopentomutase	2.28
EAMY_2304	*fruK*, fructose-1-phosphate kinase	2.28
EAMY_1212	uncharacterized protein	1.88
EAMY_3456	*glpR*, repressor of *glp* operon	1.79
EAMY_1636	*tcaB*, major facilitator superfamily transporter	1.76
EAMY_1977	uncharacterized protein	1.72
EAMY_2352	*ostA*, trehalose-6-phosphate synthase	1.57
EAMY_1143	*nagB*, glucosamine-6-phosphate isomerase	1.57
EAMY_0108	*pfkA*, 6-phosphofructokinase	1.54
EAMY_1976	*gapA*, glyceraldehyde-3-phosphate dehydrogenase A	1.53

EAMY_2102	*otsA*, trehalose-6-phosphate synthase	1.51
EAMY_1144	*nagE*, PTS system N-acetylglucosamine-specific enzyme IIC	1.48
EAMY_0631	*rpiA*, ribose 5-phosphate isomerase	1.43
EAMY_3473	*gntK*, gluconate kinase	1.31
EAMY_2056	*mdfA*, major facilitator superfamily transporter	1.27
EAMY_0118	*tpiA*, triosephosphate isomerase	1.25
EAMY_1743	major facilitator superfamily transporter	1.24
EAMY_0115	dehydrogenase	1.21
EAMY_0161	*ilvD*, dihydroxy-acid dehydratase	1.21
EAMY_2050	sugar phosphate isomerase/epimerase	1.20
EAMY_0738	*eno*, enolase	1.17
EAMY_0124	*glpX*, fructose-1,6-bisphosphatase class II	1.05
EAMY_1153	*pgm*, phosphoglucomutase	1.01
**Cell cycle control, cell division, chromosome partitioning**		
EAMY_2803	*yacK*, multicopper oxidase	2.62
EAMY_1387	*sulA*, cell division inhibitor	2.19
EAMY_1925	intracellular septation protein A	1.80
EAMY_1357	*mukF*, chromosome partition protein	1.75
EAMY_2278	*mrp*, ATPases involved in chromosome partitioning	1.36
EAMY_1358	*mukE*, chromosome partition protein	1.27
EAMY_0129	*yiiU*, 1-phosphatidylinositol-4,5-bisphosphate phosphodiesterase beta 2	1.20
EAMY_2482	*zipA*, cell division protein	1.12
**Cell motility**		
EAMY_2090	methyl-accepting chemotaxis protein	2.18
EAMY_2088	*cheB*, chemotaxis regulator	2.10
EAMY_2089	*cheR*, methyl-accepting chemotaxis protein methyltransferase	1.44
**Cell wall/membrane/envelope biogenesis**		
EAMY_2803	*yacK*, multicopper oxidase	2.62
EAMY_2614	*N*-acetylmuramic acid 6-phosphate etherase	2.47
EAMY_0323	*yrbF*, ABC transporter ATP-binding protein	2.42
EAMY_1522	transmembrane protein	2.36
EAMY_0324	*yrbE*, inner membrane protein	2.34
EAMY_2553	nucleoside-diphosphate-sugar epimerases	2.13
EAMY_1523	*ycfU*, transmembrane protein	1.93
EAMY_2883	*lpxC*, UDP-3-O-acyl N-acetylglucosamine deacetylase	1.84
EAMY_1277	*ompX*, outer membrane X	1.79
EAMY_1702	*slyB*, outer membrane lipoprotein	1.73
EAMY_0249	*ompT*, outer membrane protease	1.68
EAMY_2453	*ybdG*, small-conductance mechanosensitive channel	1.67
EAMY_0228	*murB*, UDP-N-acetylmuramate dehydrogenase	1.58

EAMY_0154	*murI*, glutamate racemase	1.42
EAMY_2705	*tsx*, nucleoside-binding outer membrane protein	1.40
EAMY_0329	*murA*, UDP-N-acetylglucosamine enolpyruvyl transferase	1.38
EAMY_2784	*mrcB*, peptidoglycan synthetase	1.32
EAMY_2531	*dapA*, dihydrodipicolinate synthase	1.32
EAMY_1364	*ompN*, outer membrane protein porin	1.30
EAMY_0716	*mltA*, membrane-bound lytic murein transglycosylase	1.26
EAMY_2747	*lpxA*, acyl-ACP-UDP-N-acetylglucosamineacetyltransferase	1.25
EAMY_1336	*lolA*, outer membrane lipoprotein-sorting protein	1.19
EAMY_0082	*kdtA*, 3-deoxy-D-manno-octulosonic-acid transferase	1.16
EAMY_2219	*yeeZ*, dTDP-glucose 4,6-dehydratase	1.10
EAMY_2961	*slt*, soluble lytic murein transglycosylase	1.09
EAMY_0620	*yggB*, small-conductance mechanosensitive channel	1.07
EAMY_1901	hypothetical protein	1.06
EAMY_1547	*ycgQ*, muramoyltetrapeptide carboxypeptidase	1.06
EAMY_1449	*mviN*, integral membrane protein	1.05
EAMY_1226	uncharacterized protein	1.03
**Coenzyme transport and metabolism**		
EAMY_0603	*metK*, *S*-adenosylmethionine synthetase	4.32
EAMY_1915	*trpE*, anthranilate synthase component I	3.67
EAMY_0158	*ilvG*, acetolactate synthase isozyme III large subunit	3.60
EAMY_1916	*trpG*, anthranilate synthase component II	2.73
EAMY_2929	*folA*, dihydrofolate reductase	2.56
EAMY_0160	*ilvE*, branched-chain amino acidaminotransferase	2.17
EAMY_2771	*hemL*, glutamate-1-semialdehyde 2,1-aminomutase	2.07
EAMY_0075	*coaBC*, phosphopantothenoylcysteinesynthetase/decarboxylase	1.99
EAMY_1606	*hemA*, glutamyl-tRNA reductase	1.97
EAMY_1750	flavoprotein monooxygenase	1.96
EAMY_1212	uncharacterized protein	1.88
EAMY_1287	*moeA*, molybdopterin biosynthesis enzyme	1.82
EAMY_0967	*thiI*, thiamine biosynthesis ATP pyrophosphatase	1.73
EAMY_1366	*pncB*, nicotinate phosphoribosyltransferase	1.65
EAMY_0752	*cysG*, uroporphyrin-III C-methyltransferase	1.59
EAMY_2292	*folE*, GTP cyclohydrolase I	1.58
EAMY_2107	*hemB*, delta-aminolevulinic acid dehydratase	1.52
EAMY_1706	*pdxH*, pyridoxinephosphate oxidase	1.48
EAMY_1802	alkanesulfonate monooxygenase	1.45
EAMY_0220	*ubiD*, 3-octaprenyl-4-hydroxybenzoate carboxy-lyase	1.42
EAMY_2413	*pdxB*, erythronate-4-phosphate dehyrogenase	1.38
EAMY_1356	*smtA*, methylase involved in ubiquinone/menaquinone biosynthesis	1.34

EAMY_2346	*ubiG*, 3-demethylubiquinone-9 3-methyltransferase	1.32
EAMY_1056	*folD*, tetrahydrofolate dehydrogenase/cyclohydrolase	1.32
EAMY_3416	*pabA*, glutamine amidotransferase	1.31
EAMY_2832	putative ABC transport system inner membrane component	1.31
EAMY_2407	*folC*, folylpolyglutamate synthase	1.30
EAMY_0969	*abpA*, ketopantoate reductase	1.29
EAMY_3684	*yieE*, phosphopantetheinyl transferase	1.28
EAMY_0753	*cysD*, sulfate adenylyltransferase subunit II	1.23
EAMY_0959	*ribD*, pyrimidine deaminase	1.21
EAMY_1112	*lipA*, lipoate synthase	1.20
EAMY_1904	*ribA*, GTP cyclohydrolase II	1.19
EAMY_1213	*moaA*, molybdenum cofactor biosynthesis enzyme	1.17
EAMY_2831	hypothetical protein	1.15
EAMY_0962	*thiL*, thiamine monophosphate kinase	1.15
EAMY_1151	*ybfF*, hydrolase	1.14
EAMY_0356	*yhbW*, alkanal monooxygenase subunit alpha	1.13
EAMY_0965	*ispA*, geranyltranstransferase	1.12
EAMY_3330	*ubiA*, 4-hydroxybenzoate-octaprenyltransferase	1.09
EAMY_1214	*moaB*, molybdopterin biosynthesis enzyme	1.09
EAMY_1635	*nadE*, NAD synthase	1.07
EAMY_0256	*hemE*, uroporphyrinogen decarboxylase	1.06
**Defense mechanisms**		
EAMY_1829	uncharacterized protein	2.26
EAMY_0947	*ahpC*, peroxiredoxin	1.97
EAMY_3326	*dinF*, sodium-driven multidrug efflux pump	1.62
EAMY_0074	*dut*, deoxyuridine 5'-triphosphate nucleotidohydrolase	1.60
EAMY_3315	uncharacterized protein	1.44
EAMY_0292	*aaeA*, *p*-hydroxybenzoic acid efflux pump subunit	1.33
EAMY_2820	*ygcB*, CRISPR-associated helicase Cas3	1.22
EAMY_1656	*btuE*, glutathione peroxidase	1.18
EAMY_2959	uncharacterized protein	1.18
EAMY_2644	hypothetical protein	1.16
EAMY_1350	*msbA*, ABC-type multidrug transport system	1.13
EAMY_1032	*ushA*, 5'-nucleotidase/2',3'-cyclic phosphodiesterase	1.02
**Energy production and conversion**		
EAMY_0159	*ilvM*, acetolactate synthase isozyme II small subunit	2.69
EAMY_1750	flavoprotein monooxygenase	1.96
EAMY_0001	*mioC*, sulfite reductase	1.84
EAMY_2907	*leuB*, 2-isopropylmalate synthase	1.73
EAMY_1537	*icdA*, isocitrate dehydrogenase	1.68
EAMY_1330	*cydD*, cysteine ABC transport system	1.57

EAMY_1165	*sdhD*, succinate dehydrogenase hydrophobic anchor subunit	1.56
EAMY_1164	*sdhC*, succinate dehydrogenase cytochrome b556	1.55
EAMY_2809	*acnB*, aconitate hydratase 2	1.55
EAMY_2941	*nhaA*, sodium/proton antiporter	1.52
EAMY_2586	malate/*L*-lactate dehydrogenases	1.41
EAMY_1714	*betB*, NAD-dependent aldehyde dehydrogenases	1.37
EAMY_1166	*sdhA*, succinate dehydrogenase flavoprotein subunit	1.33
EAMY_2835	*lpdA*, dihydrolipoamide dehydrogenase	1.33
EAMY_1373	*ssuE*, NAD(P)H-dependent FMN reductase	1.28
EAMY_1721	*rnfB*, electron transport complex protein	1.21
EAMY_2830	*qor*, NADPH:quinone reductase	1.13
EAMY_1720	*rnfC*, electron transport complex protein	1.06
EAMY_1558	*fumB*, fumarate hydratase	1.05
EAMY_1722	*rnfA*, electron transport complex protein	1.02
EAMY_2834	short-chain alcohol dehydrogenase	1.01
EAMY_3543	*gor*, glutathione oxidoreductase	1.01
**Extracellular structures**		
EAMY_2582	*yfgB*, Fe-S-cluster redox enzyme	1.15
**General function prediction only**		
EAMY_1258	*hutF*, formiminoglutamate deiminase	4.75
EAMY_1640	membrane protein	3.29
EAMY_1434	*yceA*, sulfurtransferase	2.48
EAMY_0645	Predicted hydrolases of HD superfamily	2.22
EAMY_1828	hypothetical protein	2.08
EAMY_0644	*ygfF*, oxidoreductase	1.89
EAMY_3549	*vanA*, vanillate *O*-demethylase oxygenase	1.69
EAMY_1285	*ybiT*, DrugRA2 family transporter	1.67
EAMY_2154	uncharacterized protein	1.64
EAMY_0726	uncharacterized protein	1.59
EAMY_1793	membrane protein	1.56
EAMY_1802	alkanesulfonate monooxygenase	1.45
EAMY_3181	*fxsA*, suppressor of F exclusion of phageT7	1.38
EAMY_2413	*pdxB*, erythronate-4-phosphate dehyrogenase	1.38
EAMY_2833	ABC-type spermidine/putrescine transport system	1.35
EAMY_2528	uncharacterized protein	1.34
EAMY_1913	*yciV*, phosphatase	1.31
EAMY_2575	*engA*, GTP-binding protein	1.29
EAMY_0306	*sspB*, stringent starvation protein B	1.27
EAMY_2479	*yfeH*, Predicted sodium-dependent transporter	1.27
EAMY_2351	hypothetical protein	1.27
EAMY_3332	*yjbA*, phosphate starvation-inducible E	1.17

EAMY_1400	*yccA*, membrane protein	1.14
EAMY_1151	*ybfF*, hydrolase	1.14
EAMY_2398	*yfcH*, nucleoside-diphosphate sugar epimerase	1.14
EAMY_2830	*qor*, NADPH:quinone reductase	1.13
EAMY_0356	*yhbW*, alkanal monooxygenase subunit alpha	1.13
EAMY_1928	*yciI*, dimeric alpha-beta barrel protein	1.09
EAMY_0591	*yggS*, pyridoxal phosphate-dependent enzyme	1.08
EAMY_3402	*yheS*, ATPase components of various ABC-type transport systems	1.06
EAMY_2271	*yegS*, lipid kinase	1.06
EAMY_1017	uncharacterized protein	1.04
EAMY_1300	*ybjL*, AAE family transporter	1.02
EAMY_0289	*tldD*, protease	1.01
**Inorganic ion transport and metabolism**		
EAMY_3590	*mgtA*, magnesium-transporting ATPase	3.46
EAMY_0996	*amtB*, ammonia transporter	3.26
EAMY_0487	*ftnA*, ferritin heavy chain	3.15
EAMY_1037	*ybaR*, copper-translocating P-type ATPase	2.96
EAMY_2803	*yacK*, multicopper oxidase	2.62
EAMY_2585	*sseA*, thiosulfate sulfur transferase	2.41
EAMY_3097	*ygjT*, TerC-family transporter	2.12
EAMY_3586	*yebN*, YebN family transporter	2.00
EAMY_0195	*pldA*, outer membrane phospholipase A	1.93
EAMY_3500	*zntA*, heavy metal-transporting ATPase	1.85
EAMY_1859	*ydaN*, Zinc transport protein	1.75
EAMY_0109	*sbp*, sulphate-binding protein	1.73
EAMY_3549	*vanA*, vanillate *O*-demethylase oxygenase	1.69
EAMY_3458	*glpE*, rhodanese-related sulfurtransferase	1.60
EAMY_0486	*yiuA*, ABC transporter substrate-binding protein	1.55
EAMY_2941	*nhaA*, sodium/proton antiporter	1.52
EAMY_2833	ABC-type spermidine/putrescine transport system	1.35
EAMY_0582	*yggX*, Fe-S cluster protector protein	1.33
EAMY_1198	*modA*, molybdate ABC transporter substrate-binding protein	1.33
EAMY_1073	*ophB*, ABC transporter	1.13
EAMY_2474	*yrhG*, formate/nitrite family transporter	1.12
EAMY_0424	uncharacterized protein	1.06
EAMY_3282	*yjcE*, sodium/hydrogen exchanger	1.04
**Intracellular trafficking, secretion and vesicular transport**		
EAMY_0815	*ffh*, signal recognition particle GTPase	2.10
EAMY_0646	*yqfA*, HlyIII family-channel protein	1.51
EAMY_0681	*fhaC*, hemolysin activation/secretion protein	1.10
**Lipid transport and metabolism**		

EAMY_2423	*fabB*, 3-oxoacyl-(acyl-carrier-protein) synthase	2.38
EAMY_0644	*ygfF*, oxidoreductase	1.89
EAMY_2408	*accD*, acetyl-CoA carboxylase beta subunit	1.86
EAMY_2748	*fabZ*, 3-hydroxymyristoyl/3-hydroxydecanoyl-(acyl carrier protein) dehydratases	1.46
EAMY_0200	*pldB*, lysophospholipase	1.42
EAMY_1757	uncharacterized protein	1.42
EAMY_1931	*cls*, cardiolipin synthase	1.37
EAMY_0760	*ispF*, 2-C-methyl-D-erythritol 2,4-cyclodiphosphate synthase	1.35
EAMY_0948	*yajB*, acyl carrier protein phosphodiesterase	1.20
EAMY_1656	*btuE*, glutathione peroxidase	1.18
EAMY_1926	*yciA*, acyl-CoA hydrolase	1.11
EAMY_2395	*yfcD*, NTP pyrophosphohydrolases	1.11
EAMY_0421	uncharacterized protein	1.08
EAMY_0326	hypothetical protein	1.08
EAMY_2271	*yegS*, lipid kinase	1.06
EAMY_0759	*ispD*, 4-diphosphocytidyl-2-methyl-D-erithritolsynthase	1.02
**Nucleotide transport and metabolism**		
EAMY_1258	*hutF*, formiminoglutamate deiminase	4.75
EAMY_2568	*guaB*, inosine-5'-monophosphate dehydrogenase	3.02
EAMY_2978	*deoD*, uridine phosphorylase	2.41
EAMY_1900	*pyrF*, orotidine-5'-phosphate decarboxylase	2.24
EAMY_0645	Predicted hydrolases of HD superfamily	2.22
EAMY_0737	*pyrG*, CTP synthase	2.20
EAMY_2932	*carA*, carbamoyl-phosphate synthase small subunit	2.01
EAMY_0884	*gpt*, xanthine phosphoribosyltransferase	1.97
EAMY_2283	*cdd*, cytidine deaminase	1.96
EAMY_0366	*pyrB*, aspartate carbamoyltransferase	1.92
EAMY_2052	*purT*, phosphoribosylglycinamide formyltransferase II	1.86
EAMY_2982	*deoC*, deoxyribose-phosphate aldolase	1.83
EAMY_2542	*purM*, phosphoribosylformylglycinamidine cyclo-ligase	1.81
EAMY_2567	*guaA*, GMP synthase	1.74
EAMY_2980	*deoA*, thymidine phosphorylase	1.71
EAMY_0864	*pucG*, serine-pyruvate aminotransferase	1.68
EAMY_2859	*guaC*, GMP reductase	1.63
EAMY_0074	*dut*, deoxyuridine 5'-triphosphate nucleotidohydrolase	1.60
EAMY_2610	*purl*, FGAM synthase	1.58
EAMY_0210	*udp*, uridine phosphorylase	1.54
EAMY_2529	*purC*, SAICAR synthase	1.48
EAMY_0365	*pyrI*, aspartate carbamoyltransferase	1.37
EAMY_0262	*purH*, bifunctional purine biosynthesis protein	1.36

EAMY_2931	*carB*, carbamoyl-phosphate synthase large chain	1.33
EAMY_1965	*purU*, formyltetrahydrofolate deformylase	1.24
EAMY_2959	uncharacterized protein	1.18
EAMY_2473	*nupC*, sodium/nucleoside cotransporter	1.15
EAMY_1346	*cmK*, cytidylate kinase	1.14
EAMY_1261	deoxyribonucleotidase	1.13
EAMY_2257	*udk*, uridine kinase	1.11
EAMY_2543	*purN*, phosphoribosylglycinamide formyltransferase	1.09
EAMY_0261	*purD*, phosphoribosylamine-glycine ligase	1.08
EAMY_2767	*pfs*, purine-nucleoside phosphorylase	1.07
EAMY_1050	*purE*, phosphoribosylaminoimidazole carboxylase	1.06
EAMY_2256	*dcd*, deoxycytidine triphosphate deaminase	1.06
EAMY_1032	*ushA*, 5'-nucleotidase/2',3'-cyclic phosphodiesterase	1.02
EAMY_1603	*prsA*, phosphoribosylpyrophosphate synthetase	1.00
EAMY_0589	*yggV*, xanthosine triphosphate pyrophosphatase	1.00
**Post-translational modification, protein turnover and chaperones**		
EAMY_1691	*ydhD*, glutaredoxin-related protein	2.31
EAMY_3180	*groE*, molecular chaperone	2.22
EAMY_3457	*glpG*, protein of glp regulon	1.99
EAMY_1396	hypothetical protein	1.91
EAMY_0132	*hslV*, ATP-dependent protease	1.88
EAMY_1019	*htpG*, molecular chaperone	1.81
EAMY_3179	*groL*, molecular chaperone	1.81
EAMY_1975	*yeaA*, methionine sulphoxide reductase B	1.79
EAMY_0131	*hslU*, ATP-dependent protease	1.69
EAMY_1633	*spy*, spheroplast protein Y	1.67
EAMY_3417	*ppiA*, peptidyl-prolyl *cis-trans* isomerase	1.66
EAMY_1330	*cydD*, cysteine ABC transport system	1.57
EAMY_2639	*grpE*, molecular chaperone	1.55
EAMY_3398	*slyD*, FKBP-type peptidyl-prolyl *cis-trans* isomerase	1.53
EAMY_0980	*tig*, trigger factor	1.51
EAMY_2922	*djlA*, DnaJ-like protein	1.49
EAMY_2943	*dnaJ*, molecular chaperone	1.48
EAMY_1675	*sufA*, FeS assembly scaffold	1.41
EAMY_2022	*htpX*, heat shock protein	1.40
EAMY_2706	*speG*, acetyltransferase	1.38
EAMY_0582	*yggX*, Fe-S cluster protector protein	1.33
EAMY_0305	*sspA*, glutathione S-transferase	1.32
EAMY_1040	hypothetical protein	1.30
EAMY_2646	*smpB*, tmRNA-binding protein	1.25
EAMY_0981	*clpP*, ATP-dependent Clp protease	1.23

EAMY_1331	*trxB*, thioredoxin reductase	1.17
EAMY_2944	*dnaK*, molecular chaperone	1.17
EAMY_2991	*draG*, ADP-ribosyl-[dinitrogen reductase] hydrolase	1.17
EAMY_0763	*pcm*, protein-L-isoaspartatecarboxylmethyltransferase	1.16
EAMY_1052	*ppiB*, peptidyl-prolyl cis-trans isomerase B	1.16
EAMY_1676	*sufB*, FeS assembly protein	1.14
EAMY_0655	*dsbC*, protein-disulfide isomerase	1.11
EAMY_0987	*ybaU*, parvulin-like peptidyl-prolyl isomerase	1.09
EAMY_3453	*yhgI*, Fe-S cluster biosynthesis	1.06
**Replication, recombination and repair**		
EAMY_2641	*recN*, DNA repair protein	2.96
EAMY_2051	hypothetical protein	2.36
EAMY_3296	*ssb*, single-stranded DNA-binding protein	2.22
EAMY_0882	*dinP*, DNA polymerase IV	2.15
EAMY_0079	*mutM*, formamidopyrimidine DNA glycosylase	2.08
EAMY_0805	*recA*, recombinase A	1.97
EAMY_2919	*polB*, DNA polymerase II	1.90
EAMY_1224	*rhlE*, ATP-dependent RNA helicase	1.74
EAMY_2064	*ruvA*, Holliday junction ATP-dependent DNA helicase	1.66
EAMY_2302	*nfo*, endonuclease IV	1.66
EAMY_2345	*gyrA*, DNA gyrase A subunit	1.64
EAMY_0937	*sbcD*, DNA repair exonuclease	1.57
EAMY_1211	*uvrB*, excinuclease UvrABC subunit B	1.56
EAMY_0459	*parC*, topoisomerase IV subunit A	1.52
EAMY_0762	*surE*, acid phosphatase	1.51
EAMY_0927	*rdgC*, DNA recombination-dependent growth factor C	1.50
EAMY_0725	*exo*, 5'-3' exonuclease	1.47
EAMY_0355	*deaD*, cold-shock DEAD box protein	1.47
EAMY_1654	*ihfA*, integration host factor alpha subunit	1.45
EAMY_0844	*dnaQ*, DNA polymerase III epsilon subunit	1.36
EAMY_1854	*dbpA*, ATP-dependent RNA helicase	1.30
EAMY_3144	*priB*, primosomal replication protein N	1.24
EAMY_1251	*dinG*, ATP-dependent helicase	1.23
EAMY_2063	*ruvB*, Holliday junction ATP-dependent DNA helicase	1.18
EAMY_2990	*tag*, 3-methyladenine DNA glycosylase	1.13
EAMY_0194	*uvrD*, DNA helicase II	1.13
EAMY_1692	*rnt*, ribonuclease T	1.12
EAMY_2635	*srmB*, ATP-dependent RNA helicase	1.07
EAMY_1018	*recR*, recombination protein	1.06
EAMY_3297	*uvrA*, excinuclease ATPase subunit	1.01
**Secondary metabolites biosynthesis, transport and catabolism**		

EAMY_1259	*hutI*, imidazolonepropionase	4.93
EAMY_2423	*fabB*, 3-oxoacyl-(acyl-carrier-protein) synthase	2.38
EAMY_0644	*ygfF*, oxidoreductase	1.89
**Signal transduction mechanisms**		
EAMY_0860	ABC transporter substrate-binding protein	5.28
EAMY_0995	*glnK*, nitrogen regulatory protein PII	4.12
EAMY_0266	ABC transporter substrate-binding protein	3.47
EAMY_3327	*lexA*, SOS-response transcriptional repressors	2.95
EAMY_2024	*proQ*, activator of osmoprotectant transporter	2.35
EAMY_2090	methyl-accepting chemotaxis protein	2.18
EAMY_2088	*cheB*, chemotaxis regulator	2.10
EAMY_1531	*phoP*, two-component system response regulator	1.86
EAMY_1257	uncharacterized protein	1.70
EAMY_2089	*cheR*, methyl-accepting chemotaxis protein methyltransferase	1.44
EAMY_2342	*rcsB*, response regulator	1.42
EAMY_2606	*glnB*, nitrogen regulatory protein PII	1.39
EAMY_1314	*artI*, arginine ABC transport system	1.33
EAMY_0037	*typA*, GTP-binding protein	1.29
EAMY_1878	*pspF*, response regulator	1.29
EAMY_1877	*pspA*, phase shock protein A	1.19
EAMY_0328	uncharacterized protein	1.06
EAMY_1818	*fnr*, fumarate and nitrate reduction regulatory protein	1.05
EAMY_2507	*narP*, two-component system response regulator	1.01
**Transcription**		
EAMY_0207	*metR*, transcriptional regulator	5.47
EAMY_2186	*nac*, transcriptional regulator	3.64
EAMY_3327	*lexA*, SOS-response transcriptional repressors	2.95
EAMY_0137	*metJ*, transcriptional regulator of *met* regulon	2.41
EAMY_0911	*cspA*, cold shock protein	2.24
EAMY_1739	*ynfL*, LysR family transcriptional regulator	2.18
EAMY_2185	*cbl*, Cys-regulon transcriptional activator	2.13
EAMY_1795	*rob*, AraC-family transcriptional regulator	2.13
EAMY_0499	*ascG*, transcriptional regulator	2.01
EAMY_0056	*phnA*, alkylphosphonateutilization operon protein	2.01
EAMY_0297	*argR*, arginine repressor	1.93
EAMY_0402	hypothetical protein	1.90
EAMY_1531	*phoP*, two-component system response regulator	1.86
EAMY_3456	*glpR*, repressor of *glp* operon	1.79
EAMY_0073	*ttk*, transcriptional regulator	1.70
EAMY_2598	*iscR*, transcriptional regulator	1.67
EAMY_2615	*yfhH*, transcriptional regulator	1.60

EAMY_3492	*rpoH*, RNA polymerase sigma-32 factor	1.54
EAMY_0348	*nusA*, transcription elongation factor	1.46
EAMY_2342	*rcsB*, response regulator	1.42
EAMY_0846	*gntR*, transcriptional regulator	1.35
EAMY_0958	*ybaD*, transcriptional repressor	1.35
EAMY_1278	*mntR*, transcriptional regulator	1.31
EAMY_1794	Transcriptional regulator	1.29
EAMY_1878	*pspF*, response regulator	1.29
EAMY_0270	*fis*, DNA-binding protein	1.27
EAMY_1877	*pspA*, phase shock protein A	1.19
EAMY_2717	*emrR*, transcriptional regulator	1.17
EAMY_3550	Transcriptional regulator	1.17
EAMY_3646	*yqeI*, Transcriptional regulator	1.13
EAMY_0163	*ilvY*, transcriptional regulator	1.12
EAMY_1256	*hutC*, transcriptional regulator	1.09
EAMY_0961	*nusB*, transcription termination factor	1.04
EAMY_0012	*alsR*, transcriptional regulator	1.04
EAMY_0721	*gcvA*, transcriptional regulator	1.04
EAMY_3528	AraC family transcriptional regulator	1.01
EAMY_2507	*narP*, two-component system response regulator	1.01
**Translation, ribosomal structure and biogenesis**		
EAMY_0304	*rpsI*, ribosomal protein S9	2.86
EAMY_0303	*rplM*, ribosomal protein L13	2.70
EAMY_2320	*rplY*, ribosomal protein L25	2.65
EAMY_0136	*rpmE*, ribosomal protein L31	2.64
EAMY_0071	*rph*, ribonuclease PH	2.57
EAMY_0352	*rpsO*, ribosomal protein S15	2.51
EAMY_0078	*rpmG*, ribosomal protein L33	2.43
EAMY_0232	*tufA*, translation elongation factors	2.42
EAMY_1597	*ychF*, probable translation factor	2.30
EAMY_0658	*prfB*, peptide chain release factor I	2.28
EAMY_0417	*rpsU*, ribosomal protein S21	2.12
EAMY_2069	*aspS*, aspartyl-tRNA synthetase	1.99
EAMY_3375	*rplX*, ribosomal protein L24	1.93
EAMY_3376	*rplN*, ribosomal protein L14	1.93
EAMY_0659	*lysS*, lysyl-tRNA synthetase	1.91
EAMY_3374	*rplE*, ribosomal protein L5	1.90
EAMY_2940	*rpsT*, ribosomal protein S20	1.84
EAMY_2760	*rpsB*, ribosomal protein S2	1.82
EAMY_0236	*rplA*, ribosomal protein L1	1.81
EAMY_3368	*rpmD*, ribosomal protein L30	1.80

EAMY_1467	*rluC*, pseudouridylate synthase	1.77
EAMY_0585	*yggH*, tRNA (guanine-N7-)-methyltransferase	1.76
EAMY_3373	*rpsN*, ribosomal protein S14	1.76
EAMY_0077	*rpmB*, ribosomal protein L28	1.76
EAMY_2424	uncharacterized protein	1.75
EAMY_3371	*rplF*, ribosomal protein L9	1.75
EAMY_0967	*thiI*, thiamine biosynthesis ATP pyrophosphatase	1.73
EAMY_3372	*rpsH*, ribosomal protein S8	1.72
EAMY_2497	*ypeA*, acetyltransferase	1.70
EAMY_0235	*rplK*, ribosomal protein L11	1.68
EAMY_2785	*hrpB*, ATP-dependent RNA helicase	1.68
EAMY_2758	*tsf*, elongation factor	1.67
EAMY_1534	*ycfB*, tRNA (5-methylaminomethyl-2-thiouridylate)-methyltransferase	1.65
EAMY_3633	*glyQ*, glycine tRNA synthetase alpha subunit	1.64
EAMY_0333	*rpmA*, ribosomal protein L27	1.64
EAMY_3033	*rsmC*, ribosomal RNA small subunit methyltransferase	1.62
EAMY_3370	*rplR*, ribosomal protein L18	1.60
EAMY_3369	*rpsE*, ribosomal protein S5	1.57
EAMY_3367	*rplO*, ribosomal protein L15	1.54
EAMY_2430	*yfcB*, methylase of polypeptide chain release factors	1.52
EAMY_1327	*infA*, translation initiation factor IF-1	1.52
EAMY_0734	*ygcA*, putative SAM-dependent methyltransferase	1.49
EAMY_1377	hypothetical protein	1.45
EAMY_0419	*ygjD*, putative O-sialoglycoprotein endopeptidase	1.44
EAMY_1035	*ybaK*, prolyl-tRNA synthetase	1.43
EAMY_3142	*rplI*, ribosomal protein L9	1.40
EAMY_0347	uncharacterized protein	1.39
EAMY_2706	*speG*, acetyltransferase	1.38
EAMY_2645	*yfjG*, oligoketide cyclase/lipid transport protein	1.38
EAMY_2082	*argS*, arginyl-tRNA synthetase	1.37
EAMY_3540	*yhiR*, protein involved in catabolism of external DNA	1.37
EAMY_0332	*rplU*, ribosomal protein L21	1.36
EAMY_2786	*yadP*, 2'-5' RNA ligase	1.36
EAMY_1053	*cysS*, cysteine tRNA synthetase	1.35
EAMY_0368	uncharacterized protein	1.32
EAMY_3425	*trpS*, tryptophanyl-tRNA synthetase	1.31
EAMY_3143	*rpsR*, ribosomal protein S18	1.30
EAMY_2740	*mesJ*, cell cycle protein	1.29
EAMY_2624	*lepA*, GTP-binding protein	1.28
EAMY_3360	*rplQ*, ribosomal protein L17	1.27

EAMY_1607	*prfA*, protein chain release factor B	1.27
EAMY_3390	*rpsG*, ribosomal protein S7	1.26
EAMY_0727	*yqcD*, NADPH-dependent 7-cyano-7-deazaguanine reductase	1.25
EAMY_1365	*asnS*, asparaginyl-tRNA synthetase	1.25
EAMY_3387	*rpsJ*, ribosomal protein S10	1.24
EAMY_1055	uncharacterized protein	1.24
EAMY_2733	*proS*, prolyl-tRNA synthetase	1.24
EAMY_2634	*yfiC*, O-methyltransferase	1.22
EAMY_0816	*rpsP*, ribosomal protein S16	1.22
EAMY_3634	*glyS*, glycyl-tRNA synthetase beta subunit	1.21
EAMY_3145	*rpsF*, ribosomal protein S6	1.21
EAMY_2756	*frr*, ribosome recycling factor	1.19
EAMY_1912	*yciO*, translation factor	1.19
EAMY_1536	*ymfC*, ribosomal large subunit pseudouridine synthase	1.18
EAMY_3386	*rplC*, ribosomal protein L3	1.17
EAMY_2355	hypothetical protein	1.15
EAMY_3385	*rplD*, ribosomal protein L4	1.15
EAMY_2599	*yfhQ*, rRNA methylase	1.15
EAMY_0854	*gatA*, amidase	1.14
EAMY_2732	uncharacterized protein	1.13
EAMY_3391	*rpsL*, ribosomal protein S12	1.13
EAMY_1397	*yccW*, SAM-dependent methyltransferases	1.13
EAMY_3351	*fmt*, methionyl-tRNA formyltransferase	1.12
EAMY_1845	*hrpA*, ATP-dependent helicase	1.12
EAMY_2612	*yfhC*, tRNA-specific adenosine deaminase	1.11
EAMY_2987	*prfC*, translation elongation factor	1.10
EAMY_3168	*yjeQ*, GTPase	1.10
EAMY_0817	*yfjA*, 16S rRNA-processing protein	1.09
EAMY_3352	*sun*, tRNA and rRNA cytosine-C5-methylase	1.09
EAMY_2790	*mdeA*, methionine gamma-lyase	1.08
EAMY_3389	*fusA*, Translation elongation factor	1.07
EAMY_3384	*rplW*, ribosomal protein L23	1.05
EAMY_2583	*ndk*, nucleoside diphosphate kinase	1.04
EAMY_0339	*yhbY*, CRS2-associated factor 1	1.03
EAMY_1135	*miaB*, 2-methylthioadenine synthetase	1.01
EAMY_0761	*ygbO*, tRNA pseudouridine synthase D	1.01
EAMY_0819	*rplS*, ribosomal protein L19	1.01
EAMY_0353	*pnp*, polyribonucleotide nucleotidyltransferase	1.01
**Uncharacterized/functional unknown proteins**		
EAMY_1737	hypothetical protein	5.17
EAMY_0206	hypothetical protein	5.04

EAMY_3567	hypothetical protein	4.00
EAMY_0657	hypothetical protein	3.86
EAMY_1186	hypothetical protein	3.71
EAMY_1914	hypothetical protein	3.59
EAMY_1545	*msgA*, virulence protein	3.36
EAMY_0205	hypothetical protein	3.36
EAMY_3591	*mgtC*, magnesium-transporting ATPase	2.71
EAMY_2321	hypothetical protein	2.60
EAMY_0855	hypothetical protein	2.59
EAMY_1442	*dinI*, DNA damage-inducible protein I	2.51
EAMY_1045	hypothetical protein	2.41
EAMY_1745	hypothetical protein	2.35
EAMY_1441	hypothetical protein	2.33
EAMY_2505	hypothetical protein	2.31
EAMY_3645	hypothetical protein	2.29
EAMY_0140	hypothetical protein	2.24
EAMY_0988	*ybaV*, AraC-family transcriptional regulator	2.24
EAMY_2381	hypothetical protein	2.24
EAMY_1924	*yciC*, membrane protein	2.22
EAMY_2015	hypothetical protein	2.20
EAMY_0204	hypothetical protein	2.16
EAMY_1744	hypothetical protein	2.06
EAMY_2048	hypothetical protein	2.03
EAMY_2380	hypothetical protein	1.98
EAMY_2986	*osmY*, lipoprotein	1.91
EAMY_2213	hypothetical protein	1.90
EAMY_2014	*yebH*, SAM-dependent methyltransferases	1.87
EAMY_0799	hypothetical protein	1.84
EAMY_2070	hypothetical protein	1.83
EAMY_1768	hypothetical protein	1.82
EAMY_3552	hypothetical protein	1.82
EAMY_3287	*soxS*, transcriptional regulator	1.81
EAMY_3644	Transcriptional regulator	1.81
EAMY_2044	hypothetical protein	1.80
EAMY_3589	hypothetical protein	1.78
EAMY_1544	hypothetical protein	1.76
EAMY_1492	hypothetical protein	1.73
EAMY_1044	colicin A	1.72
EAMY_1930	hypothetical protein	1.70
EAMY_2214	uncharacterized protein	1.70
EAMY_0946	hypothetical protein	1.69

EAMY_2819	*ygcL*, Cse1-family CRISPR-associated protein	1.69
EAMY_2105	*umuD*, SOS-response transcriptional repressor	1.68
EAMY_0325	uncharacterized protein	1.67
EAMY_3622	hypothetical protein	1.67
EAMY_0068	uncharacterized protein	1.67
EAMY_2043	*holE*, DNA polymerase III theta subunit	1.65
EAMY_2759	hypothetical protein	1.63
EAMY_0403	hypothetical protein	1.61
EAMY_1772	uncharacterized protein	1.61
EAMY_1974	uncharacterized protein	1.61
EAMY_2045	*exoX*, exodeoxyribonuclease X	1.60
EAMY_2605	hypothetical protein	1.59
EAMY_1634	*cho*, excinuclease	1.59
EAMY_2704	hypothetical protein	1.58
EAMY_3267	gluconate 2-dehydrogenase	1.56
EAMY_1773	*doc*, death-on-curing protein	1.55
EAMY_0291	hypothetical protein	1.53
EAMY_1067	hypothetical protein	1.53
EAMY_2496	hypothetical protein	1.52
EAMY_2736	uncharacterized protein	1.51
EAMY_2053	hypothetical protein	1.50
EAMY_2530	*nlpB*, lipoprotein 34 precursor	1.50
EAMY_0945	hypothetical protein	1.50
EAMY_2277	hypothetical protein	1.48
EAMY_2981	hypothetical protein	1.44
EAMY_3063	hypothetical protein	1.44
EAMY_1769	*yafP*, acetyltransferase	1.43
EAMY_2435	hypothetical protein	1.41
EAMY_3576	hypothetical protein	1.40
EAMY_0828	hypothetical protein	1.40
EAMY_3621	hypothetical protein	1.35
EAMY_1432	*msyB*, acidic protein	1.33
EAMY_0722	uncharacterized protein	1.33
EAMY_3064	hypothetical protein	1.33
EAMY_0011	*ygiW*, TIGR00156 family protein	1.32
EAMY_2452	hypothetical protein	1.31
EAMY_2461	hypothetical protein	1.30
EAMY_0635	uncharacterized protein	1.29
EAMY_3098	hypothetical protein	1.27
EAMY_0789	*spaT*, Type III secretion chaperone	1.27
EAMY_1317	uncharacterized protein	1.27

EAMY_2436	hypothetical protein	1.27
EAMY_2074	*yecO*, SAM-dependent methyltransferases	1.27
EAMY_2731	*rcsF*, exopolysaccharide synthesis regulator	1.27
EAMY_2939	hypothetical protein	1.26
EAMY_2047	hypothetical protein	1.26
EAMY_1415	uncharacterized protein	1.24
EAMY_2802	hypothetical protein	1.23
EAMY_1431	uncharacterized protein	1.22
EAMY_1902	*yciS*, membrane protein	1.22
EAMY_1414	hypothetical protein	1.21
EAMY_2808	hypothetical protein	1.21
EAMY_2623	*lepB*, signal peptidase I	1.20
EAMY_0586	uncharacterized protein	1.20
EAMY_1929	hypothetical protein	1.19
EAMY_0156	uncharacterized protein	1.13
EAMY_1662	uncharacterized protein	1.13
EAMY_1740	hypothetical protein	1.13
EAMY_2818	*ygcK*, Cse2-family CRISPR-associated protein	1.13
EAMY_3278	uncharacterized protein	1.11
EAMY_3493	hypothetical protein	1.11
EAMY_0978	uncharacterized protein	1.11
EAMY_2555	cold shock protein	1.11
EAMY_1535	*ymfB*, NTP pyrophosphohydrolase	1.09
EAMY_1856	lambdoid prophage Qin defective integrase	1.09
EAMY_2353	hypothetical protein	1.08
EAMY_3061	hypothetical protein	1.08
EAMY_1156	*ybgA*, membrane protein	1.08
EAMY_1246	hypothetical protein	1.06
EAMY_2049	hypothetical protein	1.06
EAMY_1033	hypothetical protein	1.06
EAMY_0660	hypothetical protein	1.05
EAMY_2075	*yecP*, SAM-dependent methyltransferase	1.05
EAMY_2091	hypothetical protein	1.05
EAMY_3289	hypothetical protein	1.04
EAMY_3058	*nuc*, phospholipase D precursor	1.04
EAMY_1108	*crcA*, outer membrane protein	1.03
EAMY_0629	uncharacterized protein	1.03
EAMY_2882	uncharacterized protein	1.03
EAMY_0517	*ykfE*, lysozyme inhibitor	1.03
EAMY_2020	uncharacterized protein	1.02
EAMY_2338	hypothetical protein	1.02

EAMY_2801	hypothetical protein	1.01
EAMY_2554	hypothetical protein	1.01
EAMY_3499	*yhhN*, membrane protein	1.00

B) Down-regulated genes in the *relA*/*spoT* mutant (adjusted *P*-value < 0.05)

Locus tag	Gene description	log_2FC
**Amino acid transport and metabolism**		
EAMY_1631	*astB*, succinylarginine dihydrolase	-3.05
EAMY_1936	*oppB*, ABC transporter permease componenet	-2.65
EAMY_1935	*oppC*, ABC transporter permease componenet	-2.43
EAMY_3609	*dppF*, ABC transporter ATP-binding protein	-2.43
EAMY_1937	*oppA*, ABC transporter periplasmic component	-2.29
EAMY_3611	*dppC*, ABC transporter	-2.23
EAMY_1934	*oppD*, ABC transporter ATPase component	-2.22
EAMY_3610	*dppD*, ABC transporter ATP-binding protein	-2.17
EAMY_3613	*dppA*, ABC transporter periplasmic component	-2.04
EAMY_3190	*asnB*, asparagine synthetase	-1.99
EAMY_3612	*dppB*, ABC transporter	-1.94
EAMY_1628	*astC*, succinylornithine transaminase	-1.88
EAMY_3485	*livG*, branched-chain amino acid ABC transport system	-1.67
EAMY_1933	*oppF*, ABC-type oligopeptide transport system	-1.63
EAMY_1629	*astA*, arginine *N*-succinyltransferase	-1.60
EAMY_1292	*yliD*, ABC-type dipeptide/oligopeptide/nickel transport system	-1.55
EAMY_1344	*serC*, 3-phosphoserine aminotransferase	-1.44
EAMY_1291	*yliC*, ABC-type dipeptide/oligopeptide/nickel transport system	-1.41
EAMY_1128	ABC-type amino acid transport system	-1.37
EAMY_2388	ABC-type spermidine/putrescine transport system	-1.36
EAMY_1127	*gltK*, ABC-type amino acid transport system	-1.35
EAMY_3486	*livG*, branched-chain amino acid ABC transport system	-1.32
EAMY_3408	threonine efflux protein	-1.29
EAMY_2590	FAD-dependent oxidoreductase	-1.26
EAMY_1126	*gltL*, ABC-type polar amino acid transport system	-1.25
EAMY_1308	*potI*, ABC-type sulfate transport system	-1.23
EAMY_2311	*yejA*, ABC-type oligopeptide transport system	-1.20
EAMY_0493	*ggt*, gamma-glutamyltransferase	-1.20
EAMY_1946	*aroQ*, 3-dehydroquinate dehydratase	-1.15
EAMY_0369	*yjgM*, histone acetyltransferase	-1.11
EAMY_0664	*lysA*, diaminopimelate decarboxylase	-1.09
EAMY_0903	*dppE*, ABC-type oligopeptide transport system	-1.07
EAMY_2763	*dapD*, tetrahydrodipicolinate N-succinyltransferase	-1.05

EAMY_3487	*livM*, ABC-type branched-chain amino acid transport system	-1.05
**Carbohydrate transport and metabolism**		
EAMY_3076	*srlA*, PTS sorbitol-specific component IIC	-2.63
EAMY_3075	*srlE*, PTS sorbitol-specific component IIB	-2.14
EAMY_3074	*srlB*, PTS sorbitol-specific component IIA	-2.07
EAMY_0912	beta-galactosidase	-1.86
EAMY_2286	*mglC*, ABC-type glucose/galactose transporter permease component	-1.82
EAMY_0372	*uhpC*, sugar phosphate permease	-1.79
EAMY_0496	*celC*, PTS system transporter subunit EIIA	-1.79
EAMY_0451	*smvA*, major facilitator superfamily transporter	-1.72
EAMY_2249	*amsB*, glycosyltransferase	-1.62
EAMY_0494	*bglA*, 6-phospho-beta-glucosidase	-1.60
EAMY_0495	*bglH*, beta-glucosidase	-1.53
EAMY_1725	*araA*, L-arabinose isomerase	-1.49
EAMY_1726	*araB*, ribulose kinase	-1.40
EAMY_3338	*pgi*, glucose-6-phosphate isomerase	-1.37
EAMY_2146	*amyA*, alpha-amylase	-1.33
EAMY_3465	*glgP*, glycogen phosphorylase	-1.31
EAMY_3635	*yahK*, alcohol dehydrogenase	-1.30
EAMY_1729	*araH*, L-arabinose transport system permease protein	-1.30
EAMY_1670	*ppsA*, phosphoenolpyruvate synthase	-1.26
EAMY_2841	*abf*, beta-xylosidase	-1.26
EAMY_1644	*yniA*, fructosamine kinase	-1.24
EAMY_1102	*ygbM*, endonuclease	-1.20
EAMY_1086	*yfbH*, xylanase/chitin deacetylase	-1.18
EAMY_1727	*araF*, ABC-type sugar transport system	-1.12
EAMY_1430	*mdoH*, membrane glycosyltransferase	-1.11
EAMY_3467	*glgC*, ADP-glucose pyrophosphorylase	-1.09
EAMY_2287	*mglA*, ABC-type sugar transport system	-1.09
EAMY_1943	*pcaK*, major facilitator superfamily permease	-1.08
EAMY_3189	major facilitator superfamily permease	-1.08
EAMY_1728	*araG*, ABC-type sugar transport system	-1.07
EAMY_3450	sucrose phosphorylase	-1.04
EAMY_3466	*glgA*, glycogen synthase	-1.01
**Cell cycle control, cell division, chromosome partitioning**		
EAMY_1992	SpoVR family protein	-1.83
**Cell motility**		
EAMY_1508	*fliI*, flagellum-specific ATP synthase	-6.25
EAMY_1509	*fliH*, flagellar assembly protein	-5.98
EAMY_1456	*flgE*, flagellar hook protein	-5.97

EAMY_1511	*fliF*, flagellar M-ring protein	-5.73
EAMY_1457	*flgF*, flagellar basal body rod protein	-5.71
EAMY_1510	*fliG*, flagellar motor switch protein	-5.66
EAMY_1453	*flgB*, flagellar basal body protein	-5.40
EAMY_1459	*flhH*, flagellar L-ring protein	-5.33
EAMY_1455	*flgD*, flagellar hook capping protein	-5.26
EAMY_1454	*flgC*, flagellar basal body rod protein	-5.23
EAMY_1507	*fliJ*, flagellar biosynthesis chaperone	-5.22
EAMY_1505	*fliL*, flagellar basal body-associated protein	-5.03
EAMY_0532	*hrcN*, type III secretion system ATPase	-5.01
EAMY_1506	*fliK*, flagellar hook-length control protein	-4.88
EAMY_1503	*fliN*, flagellar motor switch protein	-4.77
EAMY_1458	*flgG*, flagellar basal body rod protein	-4.71
EAMY_1504	*fliM*, flagellar motor switch protein	-4.66
EAMY_1460	*flgI*, flagellar P-ring protein	-4.33
EAMY_1461	*flgJ*, flagellar rod assembly protein	-4.19
EAMY_1502	*fliO*, flagellar biogenesis protein	-4.13
EAMY_2085	*flhB*, flagellar biosynthetic protein	-3.97
EAMY_1512	*fliE*, flagellar hook-basal body protein	-3.67
EAMY_1501	*fliP*, flagellar biosynthetic protein	-3.54
EAMY_2084	*flhA*, flagellar biosynthesis protein	-3.49
EAMY_1452	*flgA*, flagellar basal body P-ring biosynthesis protein	-3.30
EAMY_1500	*fliQ*, flagellar biosynthetic protein	-2.81
EAMY_2143	*fliS*, flagellin-specific chaperone	-2.08
EAMY_0475	P pilus assembly protein	-1.80
EAMY_2142	*fliD*, flagellar capping protein	-1.75
EAMY_2682	*fliR*, flagellar biosynthetic protein	-1.54
EAMY_1499	*fliR*, flagellar biosynthetic protein	-1.50
EAMY_1585	*spaO*, surface presentation of antigens protein	-1.31
EAMY_1463	*flgL*, flagellar hook-associated protein	-1.28
EAMY_1462	*flgK*, flagellar hook-associated protein	-1.25
EAMY_1582	*spaI*, Type III secretion system ATPase SpaI/InvC	-1.24
EAMY_0592	*yggR*, twitching motility protein	-1.20
EAMY_2869	*outG*, Type II secretion system protein	-1.19
EAMY_0781	*spaI*, Type III secretion system ATPase SpaI/InvC	-1.06
EAMY_2660	*flgJ*, flagellar rod assembly protein	-1.00
**Cell wall/membrane/envelope biogenesis**		
EAMY_1461	*flgJ*, flagellar rod assembly protein	-4.19
EAMY_0413	*ompU*, outer membrane protein, porin	-3.55
EAMY_0090	*wabM*, glycosyltransferase	-3.08
EAMY_2844	outer membrane protease	-2.86

EAMY_2848	outer membrane protease	-2.58
EAMY_1239	glycosyltransferase	-2.43
EAMY_2285	*sanA*, membrane protein	-1.84
EAMY_0092	*wabK*, lipopolysaccharide core biosynthesisglycosyltransferase	-1.67
EAMY_1696	*yjdB*, membrane-associated, metal-dependent hydrolase	-1.58
EAMY_2498	*amiA*, *N*-acetylmuramoyl-*L*-alanine amidase	-1.54
EAMY_1423	outer membrane protein	-1.48
EAMY_0179	*wzx*, O-antigen translocase in LPS biosynthesis	-1.45
EAMY_0900	membrane protein	-1.34
EAMY_1281	outer membrane efflux protein	-1.29
EAMY_2243	*amsK*, glycosyltransferase	-1.26
EAMY_0093	*waaC*, lipopolysaccharide heptosyltransferase-1	-1.24
EAMY_0764	*nlpD*, membrane protein	-1.23
EAMY_1086	*yfbH*, xylanase/chitin deacetylase	-1.18
EAMY_1987	*wbaP*, undecaprenyl-phosphate galactose phosphotransferase	-1.16
EAMY_0178	*wecE*, TDP-4-keto-6-deoxy-D-glucose transaminase	-1.15
EAMY_1430	*mdoH*, membrane glycosyltransferase	-1.11
EAMY_2244	*amsJ*, exopolysaccharide biosynthesis protein	-1.10
EAMY_3682	*yidC*, inner membrane protein	-1.10
EAMY_1087	*yfbI*, 4-amino-4-deoxy-L-arabinose transferase	-1.08
EAMY_3681	uncharacterized protein	-1.08
EAMY_2988	acyltransferase	-1.05
EAMY_3264	acyltransferase	-1.01
EAMY_0627	*yhjL*, oxoglutarateaminotransferase	-1.01
EAMY_2660	*flgJ*, flagellar rod assembly protein	-1.00
**Coenzyme transport and metabolism**		
EAMY_1786	*yhjG*, FAD monooxygenase	-3.65
EAMY_3194	*panE*, ketopantoate reductase	-2.52
EAMY_1206	*bioB*, biotin synthetase	-2.43
EAMY_0444	*mcyE*, Glutamate-1-semialdehyde aminotransferase	-1.90
EAMY_1121	*nadD*, nicotinic acid mononucleotide adenyltransferase	-1.89
EAMY_1207	*bioF*, 8-amino-7-oxononanoate synthase	-1.74
EAMY_2925	*pdxA*, pyridoxal phosphate biosynthesis protein	-1.63
EAMY_1208	*bioC*, biotin synthesis protein	-1.60
EAMY_3191	*panC*, panthothenate synthetase	-1.54
EAMY_0193	*yigB*, predicted hydrolase	-1.48
EAMY_1344	*serC*, 3-phosphoserine aminotransferase	-1.44
EAMY_0251	*thiE*, thiamine monophosphate synthase	-1.36
EAMY_0626	*yhjK*, HAD superfamily hydrolase	-1.32
EAMY_1563	*apbE*, thiamine biosynthesis lipoprotein	-1.32
EAMY_0972	*cyoE*, cytochrome oxidase assembly factor	-1.32

EAMY_2167	FAD-dependent monooxygenase	-1.21
EAMY_2297	*yiuC*, siderophore ABC transporter	-1.16
EAMY_0025	*mobA*, molybdopterin-guanine dinucleotide biosynthesis protein A	-1.02
**Defense mechanisms**		
EAMY_2212	*yeeO*, Na^+-driven multidrug efflux pump	-1.84
EAMY_0685	*mdtC*, multidrug reistance protein	-1.83
EAMY_1884	*sapF*, ABC-type oligopeptide transporter	-1.82
EAMY_2780	*relB*, antitoxin	-1.29
EAMY_0684	*mdtB*, cation/multidrug efflux pump	-1.26
EAMY_3578	*prtE*, membrane-fusion protein	-1.13
EAMY_2814	*ygbT*, CRISPR-associated protein Cas1	-1.09
EAMY_1068	*osmC*, peroxiredoxin	-1.06
EAMY_1422	multidrug resistance efflux pump	-1.01
**Energy production and conversion**		
EAMY_1786	*yhjG*, FAD monooxygenase	-3.65
EAMY_2349	*yfaE*, ferredoxin	-2.57
EAMY_1169	*sucB*, pyruvate/2-oxoglutarate dehydrogenase	-2.06
EAMY_1171	*sucD*, succinyl-CoA synthetase alpha subunit	-2.00
EAMY_1170	*sucC*, succinyl-CoA synthetase beta subunit	-1.98
EAMY_1630	*astD*, NAD-dependent aldehyde dehydrogenase	-1.98
EAMY_1436	*yceJ*, cytochrome b561	-1.87
EAMY_2360	*nuoN*, NADH dehydrogenase I subunit	-1.81
EAMY_1905	*acnA*, aconitate hydratase	-1.78
EAMY_1092	*cydB*, cytochrome bd-type quinol oxidase subunit II	-1.71
EAMY_1093	*cydA*, cytochrome bd-type quinol oxidase subunit I	-1.61
EAMY_3599	*dctA*, sodium/hydrogen-dicarboxylate symporters	-1.61
EAMY_1168	*sucA*, 2-oxoglutarate dehydrogenase E1 component	-1.56
EAMY_1673	*ydiJ*, FAD/FMN-containing dehydrogenase	-1.56
EAMY_2361	*nuoM*, NADH:ubiquinone oxidoreductase	-1.54
EAMY_2587	NAD-dependent aldehyde dehydrogenases	-1.51
EAMY_2362	*nuoL*, NADH:ubiquinone oxidoreductase subunit	-1.43
EAMY_2114	*aldB*, NAD-dependent aldehyde dehydrogenase	-1.39
EAMY_2365	*nuoI*, NADH dehydrogenase I chain I	-1.37
EAMY_1942	*adhE*, NAD-dependent aldehyde dehydrogenase	-1.32
EAMY_2366	*nuoH*, NADH:ubiquinone oxidoreductase subunit 1	-1.31
EAMY_2364	*nuoJ*, NADH:ubiquinone oxidoreductase subunit 6	-1.26
EAMY_1489	hypothetical protein	-1.26
EAMY_2387	*pta*, phosphate acetyltransferase	-1.21
EAMY_2167	FAD-dependent monooxygenase	-1.21
EAMY_1892	anaerobic dehydrogenase	-1.15
EAMY_2363	*nuoK*, NADH dehydrogenase I chain K	-1.10

EAMY_0973	*cyoD*, cytochrome o ubiquinol oxidase protein	-1.08
EAMY_0974	*cyoC*, cytochrome o ubiquinol oxidase subunit III	-1.07
EAMY_3248	*nemA*, NADH:flavin oxidoreductase	-1.02
EAMY_2440	*ccmH*, cytochrome c-type biogenesis protein	-1.00
**Extracellular structures**		
EAMY_0475	P pilus assembly protein	-1.80
EAMY_0592	*yggR*, twitching motility protein	-1.20
EAMY_2869	*outG*, Type II secretion system protein	-1.19
EAMY_0476	*csuC*, P pilus assembly protein	-1.05
**General function prediction only**		
EAMY_1982	hypothetical protein	-2.00
EAMY_0472	*yghA*, oxidoreductase	-1.57
EAMY_3133	*ytfP*, BtrG-like, AIG2-like family protein	-1.36
EAMY_1945	*pdc*, metal-dependent hydrolase	-1.36
EAMY_3073	*srlD*, sorbitol-6-phosphate dehydrogenase	-1.35
EAMY_0626	*yhjK*, HAD superfamily hydrolase	-1.32
EAMY_3245	*gdh*, glucose 1-dehydrogenase	-1.28
EAMY_2261	*mocA*, oxidoreductase	-1.27
EAMY_2878	*chb*, GlcNac-binding protein	-1.27
EAMY_0625	*yhjJ*, dehydrogenase	-1.24
EAMY_0837	uncharacterized protein	-1.23
EAMY_1435	hypothetical protein	-1.23
EAMY_1178	uncharacterized protein	-1.10
**Inorganic ion transport and metabolism**		
EAMY_2163	*sitD*, Mn/Zn transport system	-3.74
EAMY_2162	*sitC*, iron ABC transporter	-3.21
EAMY_2128	*ycdO*, periplasmic lipoprotein involved in iron transport	-2.79
EAMY_1936	*oppB*, ABC transporter permease componenet	-2.65
EAMY_2129	*efeU*, ferrous iron permease	-2.63
EAMY_2127	*ycdB*, iron-dependent peroxidase	-2.60
EAMY_1935	*oppC*, ABC transporter permease componenet	-2.43
EAMY_2161	*sitB*, Mn/Zn transport system	-2.28
EAMY_3611	*dppC*, ABC transporter	-2.23
EAMY_1934	*oppD*, ABC transporter ATPase component	-2.22
EAMY_3610	*dppD*, ABC transporter ATP-binding protein	-2.17
EAMY_3241	*foxR*, ferric hydroxamate receptor	-2.16
EAMY_3612	*dppB*, ABC transporter	-1.94
EAMY_3562	*sidE*, sidrophore-interacting protein	-1.93
EAMY_2927	*rdgC*, recombination-associated protein	-1.77
EAMY_3693	*pstC*, phosphate ABC transporter	-1.70
EAMY_2160	ABC-type metal ion transport system	-1.60

EAMY_3240	*dfoC*, desferrioxamine siderophore biosynthesis protein	-1.58
EAMY_1292	*yliD*, ABC-type dipeptide/oligopeptide/nickel transport system	-1.55
EAMY_0307	*cysI*, sulfite reductase alpha subunit	-1.54
EAMY_3085	*yadF*, carbonate dehydratase	-1.49
EAMY_1664	*hmuU*, iron chelate ABC transporter	-1.47
EAMY_2362	*nuoL*, NADH:ubiquinone oxidoreductase subunit	-1.43
EAMY_1291	*yliC*, ABC-type dipeptide/oligopeptide/nickel transport system	-1.41
EAMY_3084	*sul*, sulfate permease	-1.38
EAMY_1069	hypothetical protein	-1.38
EAMY_3692	*pstA*, phosphate ABC transport system	-1.17
EAMY_2297	*yiuC*, siderophore ABC transporter	-1.16
EAMY_2168	*yiuA*, ABC-type Fe3+-hydroxamate transport system	-1.14
EAMY_2472	*mntH*, manganese transport protein	-1.08
EAMY_1665	*hmuT*, iron ABC transporter substrate-binding protein	-1.08
EAMY_0903	*dppE*, ABC-type oligopeptide transport system	-1.07
EAMY_0167	*gppA*, guanosine pentaphosphatase	-1.04
**Intracellular trafficking, secretion and vesicular transport**		
EAMY_1508	*fliI*, flagellum-specific ATP synthase	-6.25
EAMY_1509	*fliH*, flagellar assembly protein	-5.98
EAMY_1511	*fliF*, flagellar M-ring protein	-5.73
EAMY_1503	*fliN*, flagellar motor switch protein	-4.77
EAMY_2143	*fliS*, flagellin-specific chaperone	-2.08
EAMY_2969	heme/hemopexin utilization protein B	-1.99
EAMY_1578	*invG*, type III secretion system outer membrane pore	-1.89
EAMY_3579	*prtD*, Type I secretion system ATPase	-1.49
EAMY_0217	*tatC*, Sec-independent protein translocase protein	-1.43
EAMY_1585	*spaO*, surface presentation of antigens protein	-1.31
EAMY_1582	*spaI*, Type III secretion system ATPase SpaI/InvC	-1.24
EAMY_0785	*spaP*, surface presentation of antigens protein	-1.22
EAMY_2869	*outG*, Type II secretion system protein	-1.19
EAMY_0781	*spaI*, Type III secretion system ATPase SpaI/InvC	-1.06
EAMY_3215	type VI secretion system core protein	-1.01
**Lipid transport and metabolism**		
EAMY_3270	*sbmA*, ABC-type long-chain fatty acid transporter	-2.15
EAMY_1427	*ymdC*, phospholipase D family protein	-2.11
EAMY_2588	opine oxidase subunit A	-1.85
EAMY_2828	*fadD*, acyl-CoA synthase	-1.83
EAMY_2827	*vraB*, 3-ketoacyl-CoA thiolase	-1.72
EAMY_1242	*cfa*, cyclopropane fatty acid synthase	-1.70
EAMY_0472	*yghA*, oxidoreductase	-1.57
EAMY_3073	*srlD*, sorbitol-6-phosphate dehydrogenase	-1.35

EAMY_0972	*cyoE*, cytochrome oxidase assembly factor	-1.32
EAMY_3245	*gdh*, glucose 1-dehydrogenase	-1.28
EAMY_0222	*fadA*, acetyl-CoA acetyltransferase	-1.22
**Nucleotide transport and metabolism**		
EAMY_2348	*nrdB*, ribonucleotide reductase	-2.54
EAMY_2113	purine-cytosine permease	-1.63
EAMY_2711	*nrdE*, ribonucleoside-diphosphate reductase subunit	-1.57
EAMY_2712	*nrdF*, ribonucleotide reductase beta subunit	-1.56
EAMY_2347	*nrdA*, ribonucleotide reductase alpha subunit	-1.54
EAMY_3192	*purD*, phosphoribosylamine-glycine ligase	-1.51
EAMY_2710	*nrdI*, ribonucleotide reductase	-1.42
EAMY_0442	dihydroorotate dehydrogenase	-1.31
EAMY_3041	nucleoside hydrolase	-1.30
EAMY_0167	*gppA*, guanosine pentaphosphatase	-1.04
**Post-translational modification, protein turnover and chaperones**		
EAMY_2296	subtilisin-like serine proteases	-1.70
EAMY_2709	*nrdH*, glutaredoxin	-1.32
EAMY_0594	*dsbD*, cytochrome c biogenesis protein	-1.32
EAMY_0915	*aspH*, membrane-bound beta-hydroxylase	-1.28
EAMY_2260	*yegD*, molecular chaperone	-1.27
EAMY_0491	*lidJ*, disulphide bond formation protein	-1.08
EAMY_2440	*ccmH*, cytochrome c-type biogenesis protein	-1.00
**Replication, recombination and repair**		
EAMY_2326	helicase	-4.20
EAMY_0047	*recG*, ATP-dependent helicase	-3.72
EAMY_0711	*recB*, exonuclease V	-2.66
EAMY_3626	*dam*, adenine-specific DNA methylase	-2.15
EAMY_0712	*recD*, exonuclease V alpha subunit	-1.65
EAMY_0850	*resB*, DNA or RNA helicase	-1.26
EAMY_1122	*holA*, DNA polymerase III delta subunit	-1.21
**Secondary metabolites biosynthesis, transport and catabolism**		
EAMY_1787	*pvcB*, pyoverdine biosynthesis protein	-3.74
EAMY_1788	*pvcA*, pyoverdine biosynthesis protein	-3.48
EAMY_2517	*entF*, non-ribosomal peptide synthetase	-2.90
EAMY_3239	*dfoA*, desferrioxamine biosynthesis protein	-1.97
EAMY_0447	*sypC*, gramicidin S synthetase II	-1.91
EAMY_2828	*fadD*, acyl-CoA synthase	-1.83
EAMY_0472	*yghA*, oxidoreductase	-1.57
EAMY_0448	*ppsD*, polyketide synthase	-1.56
EAMY_3073	*srlD*, sorbitol-6-phosphate dehydrogenase	-1.35
EAMY_3245	*gdh*, glucose 1-dehydrogenase	-1.28

EAMY_2313	*yejE*, ABC transport system	-1.17
EAMY_2821	*tauD*, taurine dioxygenase	-1.05
**Signal transduction mechanisms**		
EAMY_0049	*spoT*, GTP pyrophosphokinase	-3.09
EAMY_0735	*relA*, GTP pyrophosphokinase	-2.40
EAMY_1564	*yjdG*, two-component system response regulator	-1.77
EAMY_1565	*yjdH*, two-component system histidine kinase	-1.42
EAMY_0511	*yehT*, DNA-binding response regulator	-1.41
EAMY_0282	uncharacterized protein	-1.40
EAMY_3596	*yhjH*, FOG: EAL domain protein	-1.29
EAMY_2628	*rseC*, sigma-E factor regulatory protein	-1.27
EAMY_3569	*kdpD*, sensor histidine kinase	-1.13
EAMY_1981	*prkA*, serine protein kinase	-1.11
EAMY_3517	*rtn*, signal transduction protein containing sensor and EAL domains	-1.06
EAMY_0335	*basS*, signal transduction histidine kinase	-1.06
EAMY_0167	*gppA*, guanosine pentaphosphatase	-1.04
**Transcription**		
EAMY_0536	*hrpL*, RNA polymerase sigma factor	-5.89
EAMY_0049	*spoT*, GTP pyrophosphokinase	-3.09
EAMY_2139	*fliA*, RNA polymerase sigma factor	-2.41
EAMY_0735	*relA*, GTP pyrophosphokinase	-2.40
EAMY_0921	*iclR*, transcriptional regulator	-2.26
EAMY_0593	*sigD*, RNA polymerase sigma factor	-2.24
EAMY_1564	*yjdG*, two-component system response regulator	-1.77
EAMY_1333	*lrp*, transcriptional regulator	-1.75
EAMY_1323	cold shock protein	-1.60
EAMY_0765	*rpoS*, RNA polymerase sigma factor	-1.45
EAMY_0511	*yehT*, DNA-binding response regulator	-1.41
EAMY_0148	*oxyR*, hydrogen peroxide-inducible genes activator	-1.32
EAMY_0850	*resB*, DNA or RNA helicase	-1.26
EAMY_0373	transcription regulator	-1.25
EAMY_2592	GntR family transcriptional regulator	-1.16
EAMY_3642	*mtlR*, mannitol operon repressor	-1.13
EAMY_3072	*srlM*, sorbitol operon activator protein	-1.07
**Translation, ribosomal structure and biogenesis**		
EAMY_1382	*rmf*, ribosome modulation factor	-3.22
EAMY_3193	*hisS*, histidyl-tRNA synthetase	-2.89
EAMY_3195	*hisZ*, histidyl-tRNA synthetase	-2.20
EAMY_2926	*ksgA*, dimethyladenosine transferase	-1.53
EAMY_2228	*yeaK*, prolyl-tRNA synthetase	-1.33

EAMY_3158	*miaA*, tRNA delta(2)-isopentenylpyrophosphate transferase	-1.29
EAMY_1007	*rpmE*, ribosomal protein L31	-1.29
EAMY_1085	*yfbG*, methionyl-tRNA formyltransferase	-1.04
**Type III secretion system**		
EAMY_0542	*hrpA*, Hrp pili protein	-6.49
EAMY_0552	*hrpN*, harpin protein	-6.32
EAMY_0556	*hrpW*, harpin protein	-6.07
EAMY_0536	*hrpL*, RNA polymerase sigma factor	-5.89
EAMY_0543	*hrpB*, type III secretion system protein	-5.86
EAMY_0531	*hrpO*, type III secretion protein	-5.86
EAMY_0548	*hrpG*, type III secretion protein	-5.75
EAMY_0547	*hrpF*, type III secretion protein	-5.75
EAMY_0533	*hrpQ*, type III secretion system protein	-5.65
EAMY_0544	*hrcJ*, type III secretion inner-membrane protein	-5.42
EAMY_0534	*hrcV*, type III secretion inner-membrane protein	-5.30
EAMY_0545	*hrpD*, type III secretion protein	-5.19
EAMY_0532	*hrcN*, type III secretion system ATPase	-5.01
EAMY_0535	*hrpJ*, type III secretion system protein	-5.00
EAMY_0555	*orfC*, HrpW-specific chaperone	-4.98
EAMY_0549	*hrcC*, type III secretion system outer membrane pore	-4.70
EAMY_0557	*dspE*, Hrp secreted pathogenicity-like protein	-4.40
EAMY_0553	*orfA*, Tir chaperone family protein	-4.38
EAMY_0550	*hrpT*, type III secretion lipoprotein	-4.11
EAMY_0551	*hrpV*, type III secretion protein	-4.07
EAMY_0530	*hrpP*, type III secretion protein	-4.06
EAMY_0527	*hrcS*, type III secretion protein	-3.87
EAMY_0546	*hrpE*, type III secretion apparatus protein	-3.82
EAMY_0653	*eop2*, type III effector	-3.34
EAMY_0554	*orfB*, avirulence protein	-3.33
EAMY_0528	*hrcR*, type III secretion apparatus protein	-3.04
EAMY_0526	*hrcT*, type III secretion apparatus protein	-2.84
EAMY_0529	*hrcQ*, type III secretion system apparatus protein	-2.53
EAMY_0525	*hrcU*, type III secretion protein	-2.21
EAMY_0519	*hrpK*, pathogenicity locus protein	-2.08
EAMY_0558	*dspF*, Hrp secreted pathogenicity-like protein	-2.05
EAMY_0520	*hsvA*, Hrp-associated systemic virulence protein	-1.75
EAMY_0539	*hrpS*, sigma-54-dependent enhancer-binding protein	-1.45
EAMY_3175	*avrRpt2*, cysteine protease avirulence protein	-1.07
**Uncharacterized/functional unknown proteins**		
EAMY_2327	endonuclease	-6.19
EAMY_0389	SAM-dependent methyltransferase	-5.57

EAMY_3696	bacteriophage protein	-4.85
EAMY_1078	*inlA*, leucine-rich repeat protein	-3.95
EAMY_2849	glycosyl hydrolase	-3.83
EAMY_3625	HNH endonuclease	-3.77
EAMY_3695	*lscC*, levansucrase	-3.60
EAMY_3697	hypothetical protein	-3.57
EAMY_2845	glycosyl hydrolase	-3.49
EAMY_0524	biphenyl 2,3-dioxygenase	-3.38
EAMY_0386	cell division protein	-3.20
EAMY_0387	hypothetical protein	-3.20
EAMY_0388	*mrr*, restriction endonuclease	-3.19
EAMY_2850	dermonecrotic toxin	-3.13
EAMY_1785	*srfC*, virulence factor	-3.00
EAMY_0390	chromosome segregation ATPases	-3.00
EAMY_1426	hypothetical protein	-2.96
EAMY_1514	hypothetical protein	-2.91
EAMY_0385	hypothetical protein	-2.87
EAMY_0048	*spoU*, rRNA methylase	-2.87
EAMY_0443	hypothetical protein	-2.87
EAMY_0060	hypothetical protein	-2.80
EAMY_0769	*hpa*, lytic transglycosylase	-2.76
EAMY_1594	hypothetical protein	-2.72
EAMY_1077	hypothetical protein	-2.66
EAMY_1841	acid-shock protein	-2.66
EAMY_0686	hypothetical protein	-2.65
EAMY_0559	*rlsA*, transcriptional regulator	-2.62
EAMY_3196	ribosomal protein L11 methylase	-2.61
EAMY_1424	hypothetical protein	-2.58
EAMY_3197	*nikS*, carbamoyl-phosphate synthase large subunit	-2.56
EAMY_1309	hypothetical protein	-2.52
EAMY_2768	hypothetical protein	-2.49
EAMY_3148	hypothetical protein	-2.49
EAMY_2847	hypothetical protein	-2.48
EAMY_2112	hypothetical protein	-2.47
EAMY_1425	hypothetical protein	-2.44
EAMY_2563	hypothetical protein	-2.44
EAMY_2846	hypothetical protein	-2.44
EAMY_2843	hypothetical protein	-2.42
EAMY_0395	hypothetical protein	-2.33
EAMY_0768	hypothetical protein	-2.33
EAMY_1593	*sipD*, cell invasion protein	-2.32

EAMY_3242	hypothetical protein	-2.32
EAMY_2842	hypothetical protein	-2.32
EAMY_1784	*srfB*, virulence factor	-2.31
EAMY_2526	hypothetical protein	-2.30
EAMY_1885	hypothetical protein	-2.29
EAMY_1517	*ybcQ*, phage antitermination protein	-2.28
EAMY_3313	*ybcX*, bacteriophage tail assembly protein	-2.28
EAMY_1906	hypothetical protein	-2.28
EAMY_3689	hypothetical protein	-2.27
EAMY_0450	gramicidin S synthetase	-2.26
EAMY_3312	hypothetical protein	-2.26
EAMY_0477	hypothetical protein	-2.25
EAMY_0064	restriction endonuclease	-2.25
EAMY_0857	hypothetical protein	-2.23
EAMY_1592	*sipC*, cell invasion protein	-2.22
EAMY_1823	hypothetical protein	-2.20
EAMY_2776	ferric hydroxamate receptor	-2.19
EAMY_0059	retron reverse transcriptase	-2.19
EAMY_0918	hypothetical protein	-2.18
EAMY_2137	*yedO*, tryptophan synthase subunit beta	-2.16
EAMY_3078	*pqqF*, coenzyme PQQ synthesis protein	-2.16
EAMY_2455	hypothetical protein	-2.16
EAMY_0446	*irp*, polyketide synthase	-2.15
EAMY_2138	*fliZ*, flagellar regulatory protein	-2.15
EAMY_0474	hypothetical protein	-2.12
EAMY_3581	*prtA*, zinc-binding metalloprotease	-2.12
EAMY_2970	nuclear pore complex protein	-2.11
EAMY_1496	peptidase	-2.09
EAMY_3604	*bcsC*, cellulose synthase operon protein	-2.09
EAMY_0058	hypothetical protein	-2.08
EAMY_0091	*waaL*, O-antigen ligase	-2.08
EAMY_1004	hypothetical protein	-2.07
EAMY_0478	ferrous iron transporter	-2.06
EAMY_1091	hypothetical protein	-2.06
EAMY_0950	membrane protein	-2.06
EAMY_2451	*rcsC*, two-component system histidine kinase	-2.05
EAMY_2125	hypothetical protein	-2.03
EAMY_1310	hypothetical protein	-2.02
EAMY_1518	hypothetical protein	-2.01
EAMY_2589	ferredoxin	-1.98
EAMY_1783	*srfA*, myosin light chain kinase	-1.97

EAMY_2812	hypothetical protein	-1.96
EAMY_2179	hypothetical protein	-1.92
EAMY_2450	oxidoreductase	-1.91
EAMY_3629	hypothetical protein	-1.90
EAMY_0445	*mcyE*, beta-ketoacyl synthase	-1.90
EAMY_2144	*fliT*, flagellar export chaperone	-1.89
EAMY_1401	hypothetical protein	-1.88
EAMY_1513	hypothetical protein	-1.87
EAMY_3045	transposase	-1.87
EAMY_1579	*invE*, invasion protein	-1.87
EAMY_0672	type I addicition module toxin	-1.86
EAMY_2379	hypothetical protein	-1.82
EAMY_1638	hypothetical protein	-1.82
EAMY_3337	hypothetical protein	-1.80
EAMY_1855	hypothetical protein	-1.79
EAMY_2122	hypothetical protein	-1.79
EAMY_2145	hypothetical protein	-1.79
EAMY_3088	hypothetical protein	-1.78
EAMY_0922	hypothetical protein	-1.78
EAMY_0687	hypothetical protein	-1.77
EAMY_2952	hypothetical protein	-1.77
EAMY_2779	Toxin relE	-1.76
EAMY_1088	*yfbW*, inner membrane protein	-1.75
EAMY_3255	hypothetical protein	-1.75
EAMY_3605	*bcsB*, cellulose synthase regulator	-1.74
EAMY_0067	*int*, integrase	-1.74
EAMY_3336	hypothetical protein	-1.73
EAMY_0453	hypothetical protein	-1.72
EAMY_3533	*uspB*, universal stress protein	-1.72
EAMY_2626	*hvnA*, halovibrin	-1.72
EAMY_3055	*ogrK*, late control gene B protein	-1.71
EAMY_0512	hypothetical protein	-1.71
EAMY_2464	hypothetical protein	-1.70
EAMY_0406	superfamily I DNA and RNA helicase	-1.70
EAMY_0391	uncharacterized protein	-1.70
EAMY_1647	hypothetical protein	-1.70
EAMY_0449	polyketide synthase	-1.70
EAMY_3403	hypothetical protein	-1.70
EAMY_3238	*dfoJ*, glutamate decarboxylase	-1.69
EAMY_3580	*inh*, proteinase inhibitor	-1.68
EAMY_2330	hypothetical protein	-1.67

EAMY_1494	hypothetical protein	-1.66
EAMY_2456	hypothetical protein	-1.66
EAMY_1181	*nadA*, Quinolinate synthetase	-1.66
EAMY_1188	*rhsA*, Rhs family protein	-1.66
EAMY_0574	hypothetical protein	-1.65
EAMY_0010	hypothetical protein	-1.65
EAMY_3464	hypothetical protein	-1.64
EAMY_2777	*fhuA*, outer membrane receptor protein	-1.64
EAMY_2124	NADPH-dependent oxidoreductase	-1.63
EAMY_1842	hypothetical protein	-1.63
EAMY_2871	*outI*, Type II secretion system protein	-1.63
EAMY_0838	uncharacterized protein	-1.62
EAMY_1250	acetyltransferase	-1.61
EAMY_2189	hypothetical protein	-1.60
EAMY_0624	uncharacterized protein	-1.57
EAMY_1967	uncharacterized protein	-1.56
EAMY_1076	hypothetical protein	-1.56
EAMY_0840	*mltD*, soluble lytic murein transglycosylase	-1.56
EAMY_0046	hypothetical protein	-1.53
EAMY_1549	hypothetical protein	-1.53
EAMY_2100	*flhD*, flagellar transcriptional activator	-1.52
EAMY_1572	hypothetical protein	-1.52
EAMY_1944	uncharacterized protein	-1.52
EAMY_1335	hypothetical protein	-1.52
EAMY_3614	hypothetical protein	-1.51
EAMY_0518	uncharacterized protein	-1.51
EAMY_0839	hypothetical protein	-1.51
EAMY_2166	hypothetical protein	-1.50
EAMY_0405	hypothetical protein	-1.49
EAMY_2194	membrane protein	-1.49
EAMY_2099	*flhC*, flagellar transcriptional activator	-1.48
EAMY_1243	hypothetical protein	-1.48
EAMY_2245	*amsF*, exopolysaccharide biosynthesis protein	-1.48
EAMY_2159	hypothetical protein	-1.48
EAMY_0757	*ygbE*, inner membrane protein	-1.47
EAMY_3203	hypothetical protein	-1.47
EAMY_3319	hypothetical protein	-1.46
EAMY_3615	hypothetical protein	-1.46
EAMY_2602	*csiE*, transcriptional anti-terminator	-1.46
EAMY_1621	uncharacterized protein	-1.46
EAMY_3568	hypothetical protein	-1.45

EAMY_2076	hypothetical protein	-1.45
EAMY_0956	lipoprotein	-1.45
EAMY_1890	lipoprotein	-1.45
EAMY_2270	hypothetical protein	-1.45
EAMY_2230	glycosyl/glycerophosphate transferase	-1.44
EAMY_0608	colicin V secretion/processing ATP-binding protein	-1.44
EAMY_1891	lipoprotein	-1.44
EAMY_2274	hypothetical protein	-1.44
EAMY_3325	*yjbJ*, CsbD family protein	-1.43
EAMY_0699	hypothetical protein	-1.43
EAMY_3166	hypothetical protein	-1.43
EAMY_3582	hypothetical protein	-1.43
EAMY_1583	hypothetical protein	-1.42
EAMY_1155	hypothetical protein	-1.42
EAMY_0825	hypothetical protein	-1.41
EAMY_2992	hypothetical protein	-1.41
EAMY_3628	hypothetical protein	-1.41
EAMY_0004	uncharacterized protein	-1.40
EAMY_0957	*ygaU*, peptidoglycan-binding protein	-1.39
EAMY_3516	hypothetical protein	-1.39
EAMY_2901	hypothetical protein	-1.39
EAMY_2870	*outH*, Type II secretion system protein	-1.39
EAMY_2187	hypothetical protein	-1.38
EAMY_2409	hypothetical protein	-1.38
EAMY_2964	hypothetical protein	-1.38
EAMY_1266	hypothetical protein	-1.37
EAMY_3068	hypothetical protein	-1.37
EAMY_2337	hypothetical protein	-1.37
EAMY_1577	*invF*, AraC-type DNA-binding domain-containing protein	-1.36
EAMY_2571	hypothetical protein	-1.35
EAMY_3597	hypothetical protein	-1.35
EAMY_3217	hypothetical protein	-1.34
EAMY_1519	hypothetical protein	-1.34
EAMY_3584	hypothetical protein	-1.34
EAMY_0623	*fmo*, pyruvate/2-oxoglutarate dehydrogenase complex	-1.34
EAMY_1759	hypothetical protein	-1.34
EAMY_0540	hypothetical protein	-1.33
EAMY_1627	hypothetical protein	-1.33
EAMY_1438	hypothetical protein	-1.33
EAMY_2178	*pglA*, polygalacturonase	-1.33
EAMY_0026	uncharacterized protein	-1.33

EAMY_1146	hypothetical protein	-1.32
EAMY_1835	long tail fiber protein	-1.32
EAMY_2282	*yohK*, inner membrane protein	-1.31
EAMY_0288	uncharacterized protein	-1.31
EAMY_2993	hypothetical protein	-1.31
EAMY_3674	hypothetical protein	-1.31
EAMY_2098	hypothetical protein	-1.31
EAMY_3016	hypothetical protein	-1.30
EAMY_0404	*lcrS*, transposase and inactivated derivatives	-1.30
EAMY_1089	hypothetical protein	-1.30
EAMY_2135	*D*-cysteine desulfhydrase	-1.29
EAMY_1282	methyl-accepting chemotaxis protein	-1.29
EAMY_0393	hypothetical protein	-1.29
EAMY_1094	hypothetical protein	-1.29
EAMY_3583	hypothetical protein	-1.28
EAMY_1233	hypothetical protein	-1.28
EAMY_2825	hypothetical protein	-1.28
EAMY_1751	hypothetical protein	-1.28
EAMY_0688	*nodT*, outer membrane protein	-1.27
EAMY_3015	type VI secretion system-associated protein	-1.27
EAMY_2995	ferric aerobactin receptor	-1.27
EAMY_3310	*int*, integrase	-1.26
EAMY_0622	acyltransferase	-1.26
EAMY_2136	hypothetical protein	-1.26
EAMY_3198	hypothetical protein	-1.26
EAMY_0183	hypothetical protein	-1.25
EAMY_1095	hypothetical protein	-1.25
EAMY_3423	hypothetical protein	-1.25
EAMY_1550	hypothetical protein	-1.25
EAMY_2778	hypothetical protein	-1.25
EAMY_0609	hypothetical protein	-1.24
EAMY_2900	hypothetical protein	-1.24
EAMY_3244	hypothetical protein	-1.24
EAMY_1539	hypothetical protein	-1.24
EAMY_3355	hypothetical protein	-1.23
EAMY_1581	*invB*, surface presentation of antigen	-1.23
EAMY_2123	iodotyrosine dehalogenase I	-1.23
EAMY_3333	*ymcA*, lipoprotein	-1.23
EAMY_2132	hypothetical protein	-1.22
EAMY_3221	hypothetical protein	-1.22
EAMY_1097	hypothetical protein	-1.22

EAMY_2899	hypothetical protein	-1.22
EAMY_1361	uncharacterized protein	-1.22
EAMY_3592	hypothetical protein	-1.21
EAMY_3044	hypothetical protein	-1.21
EAMY_1840	hypothetical protein	-1.21
EAMY_0180	*wecF*, 4-alpha-*L*-fucosyltransferase	-1.20
EAMY_2994	*iutA*, ferric aerobactin receptor	-1.20
EAMY_0836	hypothetical protein	-1.20
EAMY_1697	hypothetical protein	-1.19
EAMY_0792	*spiD*, cell invasion protein	-1.19
EAMY_1129	hypothetical protein	-1.18
EAMY_2248	*amsC*, exopolysaccharide biosynthesis protein	-1.18
EAMY_1419	hypothetical protein	-1.18
EAMY_0991	*pagO*, permease of the drug/metabolite transporter (DMT) superfamily	-1.18
EAMY_0793	hypothetical protein	-1.18
EAMY_2316	hypothetical protein	-1.18
EAMY_3111	hypothetical protein	-1.17
EAMY_1820	hypothetical protein	-1.16
EAMY_0913	hypothetical protein	-1.16
EAMY_2281	*yohK*, inner membrane protein	-1.16
EAMY_3013	type VI secretion system-associated protein	-1.16
EAMY_2344	hypothetical protein	-1.16
EAMY_0456	*mdaB*, NADPH-quinone reductase	-1.16
EAMY_1645	*ydiZ*, hypothetical protein	-1.16
EAMY_0231	hypothetical protein	-1.15
EAMY_0932	hypothetical protein	-1.15
EAMY_0560	*clpB*, chaperone protein	-1.15
EAMY_0902	hypothetical protein	-1.15
EAMY_3322	hypothetical protein	-1.15
EAMY_0473	hypothetical protein	-1.15
EAMY_0770	hypothetical protein	-1.15
EAMY_3573	K^+-transporting ATPase subunit F	-1.15
EAMY_0177	*wecD*, TDP-D-fucosamine acetyltransferase	-1.15
EAMY_1249	DNA-binding protein	-1.15
EAMY_0604	hypothetical protein	-1.15
EAMY_2027	uncharacterized protein	-1.14
EAMY_1595	acyl carrier protein	-1.14
EAMY_3002	flagellar L-ring protein	-1.14
EAMY_2627	*ydfT*, antitermination protein	-1.14
EAMY_0771	*orgAa*, bacterial type III secretion apparatus protein	-1.14

EAMY_1813	hypothetical protein	-1.13
EAMY_0791	hypothetical protein	-1.13
EAMY_0573	hypothetical protein	-1.13
EAMY_1241	lipoprotein	-1.13
EAMY_0675	hypothetical protein	-1.13
EAMY_2083	*flhE*, flagellar protein	-1.12
EAMY_3017	type VI secretion system-associated protein	-1.12
EAMY_2703	hypothetical protein	-1.12
EAMY_1283	hypothetical protein	-1.12
EAMY_1898	hypothetical protein	-1.12
EAMY_0756	hypothetical protein	-1.11
EAMY_2813	*ygbF*, CRISPR-associated protein Cas2	-1.10
EAMY_2997	hypothetical protein	-1.10
EAMY_0736	pyrophosphatase	-1.09
EAMY_2457	hypothetical protein	-1.09
EAMY_1237	hypothetical protein	-1.09
EAMY_0901	hypothetical protein	-1.09
EAMY_2996	*iutA*, ferric aerobactin receptor	-1.09
EAMY_1889	lipoprotein	-1.09
EAMY_2190	excinuclease ATPase subunit	-1.08
EAMY_2998	hypothetical protein	-1.08
EAMY_2165	*ospG*, serine/threonine protein kinase	-1.08
EAMY_0575	site-specific recombinase	-1.08
EAMY_2329	transposase and inactivated derivatives	-1.08
EAMY_0057	hypothetical protein	-1.07
EAMY_0384	hypothetical protein	-1.07
EAMY_0923	hypothetical protein	-1.07
EAMY_1437	hypothetical protein	-1.07
EAMY_2177	hypothetical protein	-1.07
EAMY_0455	hypothetical protein	-1.07
EAMY_1028	hypothetical protein	-1.06
EAMY_1836	hypothetical protein	-1.05
EAMY_3276	*yjcH*, inner membrane protein	-1.04
EAMY_2625	hypothetical protein	-1.04
EAMY_3449	hypothetical protein	-1.04
EAMY_3018	type VI secretion system-associated protein	-1.03
EAMY_0541	hypothetical protein	-1.03
EAMY_0794	hypothetical protein	-1.03
EAMY_3660	hypothetical protein	-1.03
EAMY_1248	hypothetical protein	-1.02
EAMY_3300	*eaeH*, bacterial surface protein	-1.02

EAMY_0514	hypothetical protein	-1.01
EAMY_3664	hypothetical protein	-1.01
EAMY_0820	Rf1 protein	-1.01
