# Supplementary material for: The stringent response regulator (p) ppGpp mediates virulence gene expression and survival in Erwinia amylovora
Source: BMC Genomics. 2020 Mar 30;21:261. doi: 10.1186/s12864-020-6699-5 (PMC7106674; doi:10.1186/s12864-020-6699-5)
Supplement: Supplementary file 2 — Additional file 2: Table S2. List of DEGs of Ea1189 at 6 h versus at 3 h. [file 12864_2020_6699_MOESM2_ESM.pdf]

Table S1: Differentially expressed genes from RNA-seq analyses of the WT between 3 h and 6 h incubation in the *hrp*-inducing medium. Differentially expressed genes are grouped into functional categories according to the clusters of orthologous group (COG) database: FC, fold changes

A) Up-regulated genes from 3 h to 6 h (adjusted *P*-value < 0.05)

Locus tag	Gene description	log_2FC
**Amino acid transport and metabolism**		
EAMY_0208	*metE*, methionine synthase II	2.50
EAMY_0493	*ggt*, gamma-glutamyltransferase	2.44
EAMY_0141	*metF*, 5,10-methylenetetrahydrofolate reductase	1.92
EAMY_2715	*proX*, glycine betaine/choline-binding protein	1.62
EAMY_3342	*metA*, homoserine transsuccinylase	1.36
EAMY_1210	ABC-type spermidine/putrescine transport	1.34
EAMY_1072	*ophA*, ABC transporter substrate-binding protein	1.22
EAMY_1071	homocysteine *S*-methyltransferase family protein	1.16
EAMY_2728	*metN*, methionine ABC transporter ATP-binding protein	1.15
EAMY_2275	*aroQ*, chorismate mutase	1.15
EAMY_0876	*eif*, translation initiation factor EIF-2B	1.11
EAMY_0875	dioxygenase	1.10
EAMY_2714	*proW*, glycine betaine/choline-binding protein	1.06
EAMY_0874	*masA*, enolase-phosphatase	1.06
EAMY_0138	*metB*, cystathionine gamma-synthase	1.04
EAMY_1074	*ophC*, ABC transporter permease	1.03
EAMY_2713	*proV*, glycine betaine/choline-binding protein	1.01
**Carbohydrate transport and metabolism**		
EAMY_0497	*celB*, PTS system transporter subunit EIIC	3.59
EAMY_0498	*celA*, PTS system transporter subunit EIIB	3.54
EAMY_0495	*bglH*, beta-glucosidase	3.48
EAMY_0496	*celC*, PTS system transporter subunit EIIA	3.41
EAMY_0494	*bglA*, 6-phospho-beta-glucosidase	3.19
EAMY_0873	methylthioribulose-1-phosphate dehydratase	1.25
**Cell motility**		
EAMY_0532	*hrcN*, type III secretion system ATPase	1.72
**Coenzyme transport and metabolism**		
EAMY_1206	*bioB*, biotin synthetase	2.75
EAMY_1208	*bioC*, biotin synthesis protein	2.71
EAMY_1207	*bioF*, 8-amino-7-oxononanoate synthase	2.60
EAMY_1209	*bioD*, dethiobiotin synthetase	2.57
EAMY_0444	*mcyE*, Glutamate-1-semialdehyde aminotransferase	1.53
EAMY_0603	*metK*, *S*-adenosylmethionine synthetase	1.52

EAMY_0441	creatininase	1.21
EAMY_1205	*bioA*, DAPA aminotransferase	1.09
**Defense mechanisms**		
EAMY_2814	*ygbT*, CRISPR-associated protein Cas1	1.47
**Inorganic ion transport and metabolism**		
EAMY_1074	*ophC*, ABC transporter permease	1.03
**Intracellular trafficking, secretion and vesicular transport**		
EAMY_3019	type VI secretion system-associated protein	1.22
**Lipid transport and metabolism**		
EAMY_2827	*vraB*, 3-ketoacyl-CoA thiolase	1.55
EAMY_2828	*fadD*, acyl-CoA synthase	1.48
EAMY_2829	acyl-CoA thioester hydrolase	1.18
**Nucleotide transport and metabolism**		
EAMY_0442	dihydroorotate dehydrogenase	1.20
**Post-translational modification, protein turnover and chaperones**		
EAMY_0595	peroxiredoxin	2.26
EAMY_0594	*dsbD*, cytochrome c biogenesis protein	2.06
**Secondary metabolites biosynthesis, transport and catabolism**		
EAMY_2517	*entF*, non-ribosomal peptide synthetase	1.70
EAMY_2828	*fadD*, acyl-CoA synthase	1.48
EAMY_0447	*sypC*, gramicidin S synthetase II	1.37
EAMY_0448	*ppsD*, polyketide synthase	1.32
EAMY_0441	creatininase	1.21
**Transcription**		
EAMY_0593	*sigD*, RNA polymerase sigma factor	1.79
EAMY_0207	*metR*, transcriptional regulator	1.37
EAMY_0499	*ascG*, transcriptional regulator	1.14
EAMY_0536	*hrpL*, RNA polymerase sigma factor	1.04
**Type III secretion system**		
EAMY_0542	*hrpA*, Hrp pili protein	2.54
EAMY_0558	*dspF*, Hrp secreted pathogenicity-like protein	2.11
EAMY_0552	*hrpN*, harpin protein	2.11
EAMY_0527	*hrcS*, type III secretion protein	1.90
EAMY_0557	*dspE*, Hrp secreted pathogenicity-like protein	1.86
EAMY_0530	*hrpP*, type III secretion protein	1.83
EAMY_0551	*hrpV*, type III secretion protein	1.82
EAMY_0531	*hrpO*, type III secretion protein	1.78
EAMY_0529	*hrcQ*, type III secretion system apparatus protein	1.75
EAMY_0528	*hrcR*, type III secretion apparatus protein	1.75
EAMY_0555	*orfC*, HrpW-specific chaperone	1.75
EAMY_0532	*hrcN*, type III secretion system ATPase	1.72

EAMY_0547	*hrpF*, type III secretion protein	1.71
EAMY_0526	*hrcT*, type III secretion apparatus protein	1.71
EAMY_0550	*hrpT*, type III secretion lipoprotein	1.71
EAMY_0548	*hrpG*, type III secretion protein	1.71
EAMY_0544	*hrcJ*, type III secretion inner-membrane protein	1.70
EAMY_0545	*hrpD*, type III secretion protein	1.69
EAMY_0549	*hrcC*, type III secretion system outer membrane pore	1.68
EAMY_0556	*hrpW*, harpin protein	1.68
EAMY_0525	*hrcU*, type III secretion protein	1.68
EAMY_0533	*hrpQ*, type III secretion system protein	1.67
EAMY_0543	*hrpB*, type III secretion system protein	1.67
EAMY_0546	*hrpE*, type III secretion apparatus protein	1.65
EAMY_0554	*orfB*, avirulence protein	1.64
EAMY_0535	*hrpJ*, type III secretion system protein	1.63
EAMY_0553	*orfA*, Tir chaperone family protein	1.61
EAMY_0534	*hrcV*, type III secretion inner-membrane protein	1.60
EAMY_0520	*hsvA*, Hrp-associated systemic virulence protein	1.60
EAMY_0521	*hsvB*, Hrp-associated systemic virulence protein	1.40
EAMY_0519	*hrpK*, pathogenicity locus protein	1.38
EAMY_0653	*eop2*, type III effector	1.36
EAMY_0536	*hrpL*, RNA polymerase sigma factor	1.04
EAMY_0522	*hsvC*, Hrp-associated systemic virulence protein	1.02
**Uncharacterized/functional unknown proteins**		
EAMY_0524	hypothetical protein	1.68
EAMY_0446	hypothetical protein	1.52
EAMY_2816	*ygcL*, Cse1-family CRISPR-associated protein	1.51
EAMY_0443	*ygbF*, CRISPR-associated protein Cas2	1.51
EAMY_2817	*ygcK*, Cse2-family CRISPR-associated protein	1.50
EAMY_3111	*ygcH*, Cse3-family CRISPR-associated protein	1.40
EAMY_0445	*mcyE*, beta-ketoacyl synthase	1.40
EAMY_2815	hypothetical protein	1.39
EAMY_2818	*ygcJ*, Cse4-family CRISPR-associated protein	1.37
EAMY_2813	hypothetical protein	1.33
EAMY_2819	*ygcI*, CRISPR-associated protein Cas5	1.30
EAMY_0523	*irp*, polyketide synthase	1.30
EAMY_0206	biphenyl 2,3-dioxygenase	1.26

B) Down-regulated genes from 3 h to 6 h (adjusted *P*-value < 0.05)

Locus tag	Gene description	log_2FC
**Amino acid transport and metabolism**		

EAMY_1631	*astB*, succinylarginine dihydrolase	-1.27
EAMY_1628	*astC*, succinylornithine transaminase	-1.22
EAMY_1629	*astA*, arginine *N*-succinyltransferase	-1.18
EAMY_2799	*medA*, methionine gamma-lyase	-1.13
EAMY_2196	*adhB*, alcohol dehydrogenase	-1.03
**Carbohydrate transport and metabolism**		
EAMY_3043	*ydfJ*, major facilitator superfamily permease	-1.28
**Energy production and conversion**		
EAMY_1630	*astD*, NAD-dependent aldehyde dehydrogenase	-1.32
EAMY_3089	*fadH*, NADH:flavin oxidoreductase	-1.03
**General function prediction only**		
EAMY_2196	*adhB*, alcohol dehydrogenase	-1.03
**Intracellular trafficking, secretion and vesicular transport**		
EAMY_2969	heme/hemopexin utilization protein B	-1.03
**Lipid transport and metabolism**		
EAMY_0878	*yafH*, Acyl-CoA dehydrogenase	-1.35
EAMY_0223	*fadB*, fatty acid oxidation complex subunit alpha	-1.14
EAMY_0222	*fadA*, acetyl-CoA acetyltransferase	-1.08
**Uncharacterized/functional unknown proteins**		
EAMY_2970	nuclear pore complex protein	-1.09
