# Supplementary figures and images for: The stringent response regulator (p) ppGpp mediates virulence gene expression and survival in Erwinia amylovora
Source: BMC Genomics. 2020 Mar 30;21:261. doi: 10.1186/s12864-020-6699-5 (PMC7106674; doi:10.1186/s12864-020-6699-5)

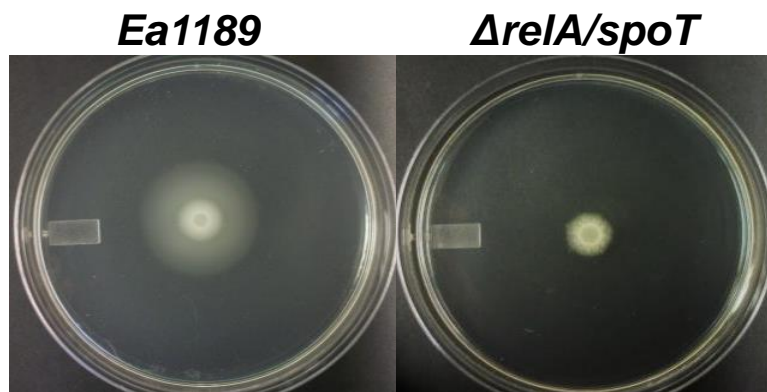

**Figure S1**

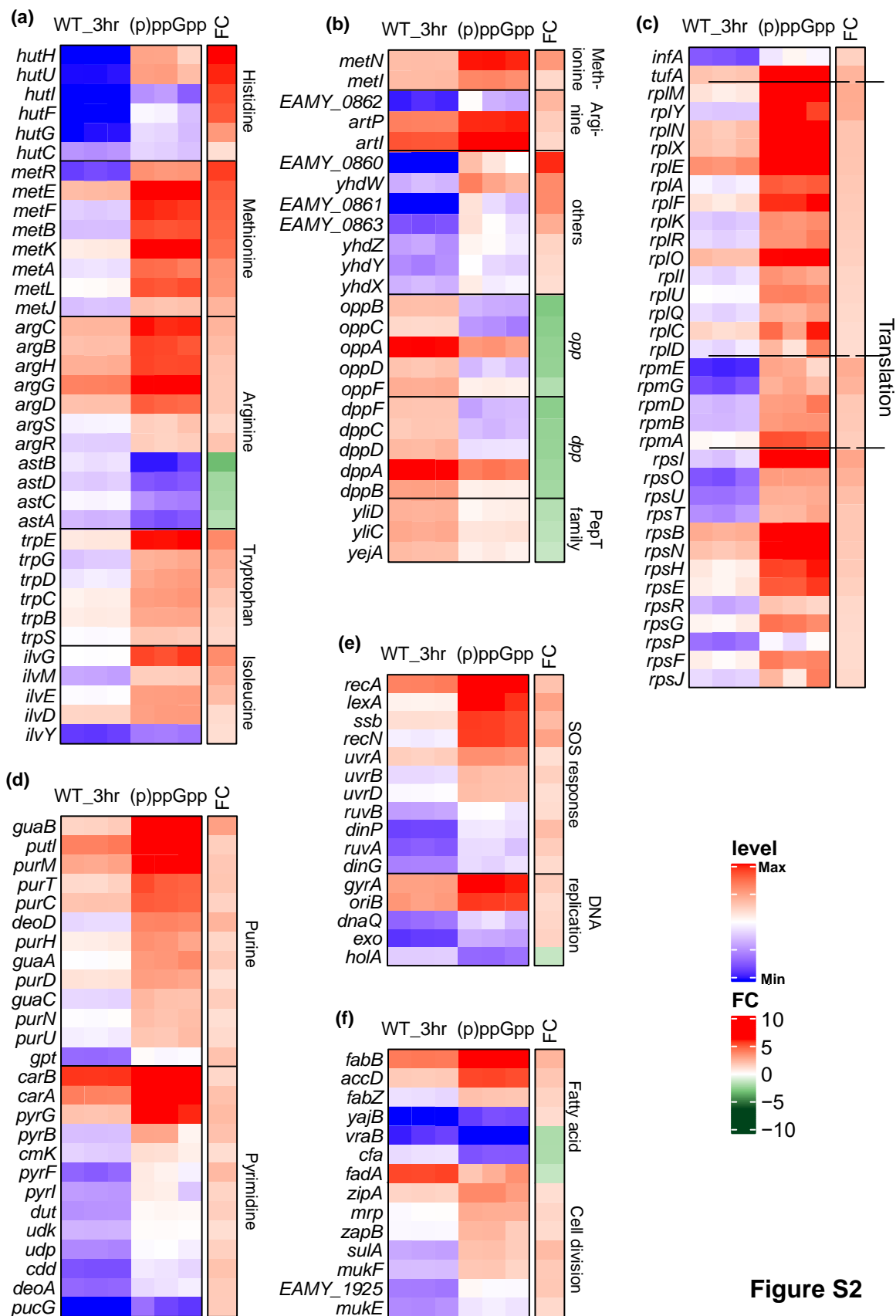

Figure S2

Supplement: Supplementary file 3 — Additional file 3: Figure S1. Motility of the wild type Ea1189 and the ∆relA/spoT mutant on soft tryptone agar plates (3%) at 28 °C and photographs were taken after 48 h. Fig. S2. Differentially expressed genes negatively regulated by (p) ppGpp. (a) amino acid biosynthesis and degradation. (b) amino acid and peptide transport systems. (c) translation (d) nucleotide metabolism. (e) DNA repair/replication (f) lipid metabolism/cell cycle. White represents mean of expression level (log2CPM), dark blue represents minimal gene expression, and bright red represents maximal gene expression. In the side bar (right), dark green represents lower negative fold change (log2FC), and bright red represents the higher positive log2FC. [file 12864_2020_6699_MOESM3_ESM.pdf]
