# Supplementary material for: The stringent response regulator (p) ppGpp mediates virulence gene expression and survival in Erwinia amylovora
Source: BMC Genomics. 2020 Mar 30;21:261. doi: 10.1186/s12864-020-6699-5 (PMC7106674; doi:10.1186/s12864-020-6699-5)
Supplement: Supplementary file 4 — Additional file 4: Table S3. Primers for qRT-PCR used in this study. [file 12864_2020_6699_MOESM4_ESM.pdf]

Table S1. Primers for qRT-PCR used in this study

Primer	Sequence (5' to 3')
EA_metA_F	GCAGACGCGCGATAGCAAAC
EA_metA_R	TCCCGTAGGGAAGTCAGCGT
EA_guaB_F	GGCGAAAACGAGTTGGTCGG
EA_guaB_R	TCCACCACCAGCGCTTTTTC
EA_sucB_F	CCGTAGCCACCTGGCACAAA
EA_sucB_R	AGAGGCCGGTACTTCCAGCA
EA_foxR_F	AAACCGGTGCTCAGTTTTCT
EA_foxR_R	CTTCGTGCCTATCTGCAAAA
EA_pvcB_F	GGGCCGTCATATCGGTGAGC
EA_pvcB_R	TAGCGGGTCAGGCTGTCACT
EA_oppB_F	AGCCGCTTCATTCCCGGTTT
EA_oppB_R	GCTTCAGCGCAGCAATCACC
EA_dppA_F	CGCCAGGGCGTGAAGTGGCA
EA_dppA_R	TTAACAATTTTGCTGATCAG
EA_rpoN_F	AAGCGGTACTGAAACGGGTA
EA_rpoN_R	GCATCAGACTGCGAAAATCA
EA_sigD_F	CGATCCACAGCGCCCCATAC
EA_sigD_R	TCCAGACATCCGCTTAACGC
EA_dksA_F	GGTGACTCACATGCAAGACG
EA_dksA_R	GATTTCAACGCCACAGGATT
EA_hrpS_F	AATGCTACGCGTGCTGGAAA
EA_hrpS_R	AACAATGGCGTTTGCGTTGC
EA_hrpL_F	TTAAGGCAATGCCAAACACC
EA_hrpL_R	GACGCGTGCATCATTTTATT
EA_hrpA_F	GAGTCCATTTTGCCATCCAG
EA_hrpA_R	TGGCAGGCAGTTCACTTACA
EA_hrpN_F	GCTTTTGCCCATGATTTGTC
EA_hrpN_R	CAACCCGTTCTTTCGTCAAT
